# Supplementary figures and images for: Estimating Transfer Entropy in Continuous Time Between Neural Spike Trains or Other Event-Based Data
Source: PLoS Comput Biol. 2021 Apr 19;17(4):e1008054. doi: 10.1371/journal.pcbi.1008054 (PMC8084348; doi:10.1371/journal.pcbi.1008054)

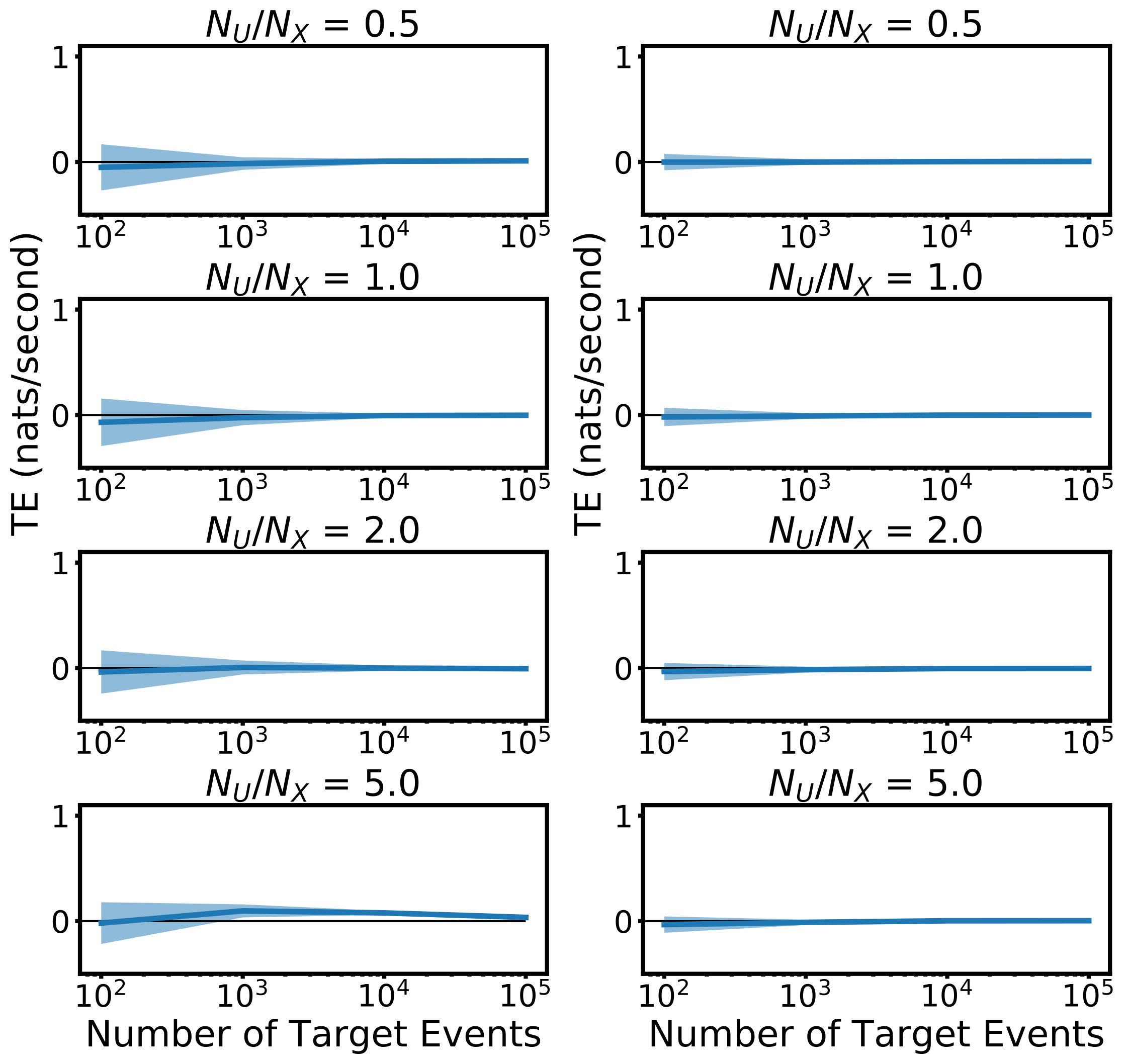

Supplement: S1 Fig — The results of an identical experimental setup to those displayed in Fig 2, but with history embedding lengths of lX = lY = 3. (TIFF) [file pcbi.1008054.s001.tiff]

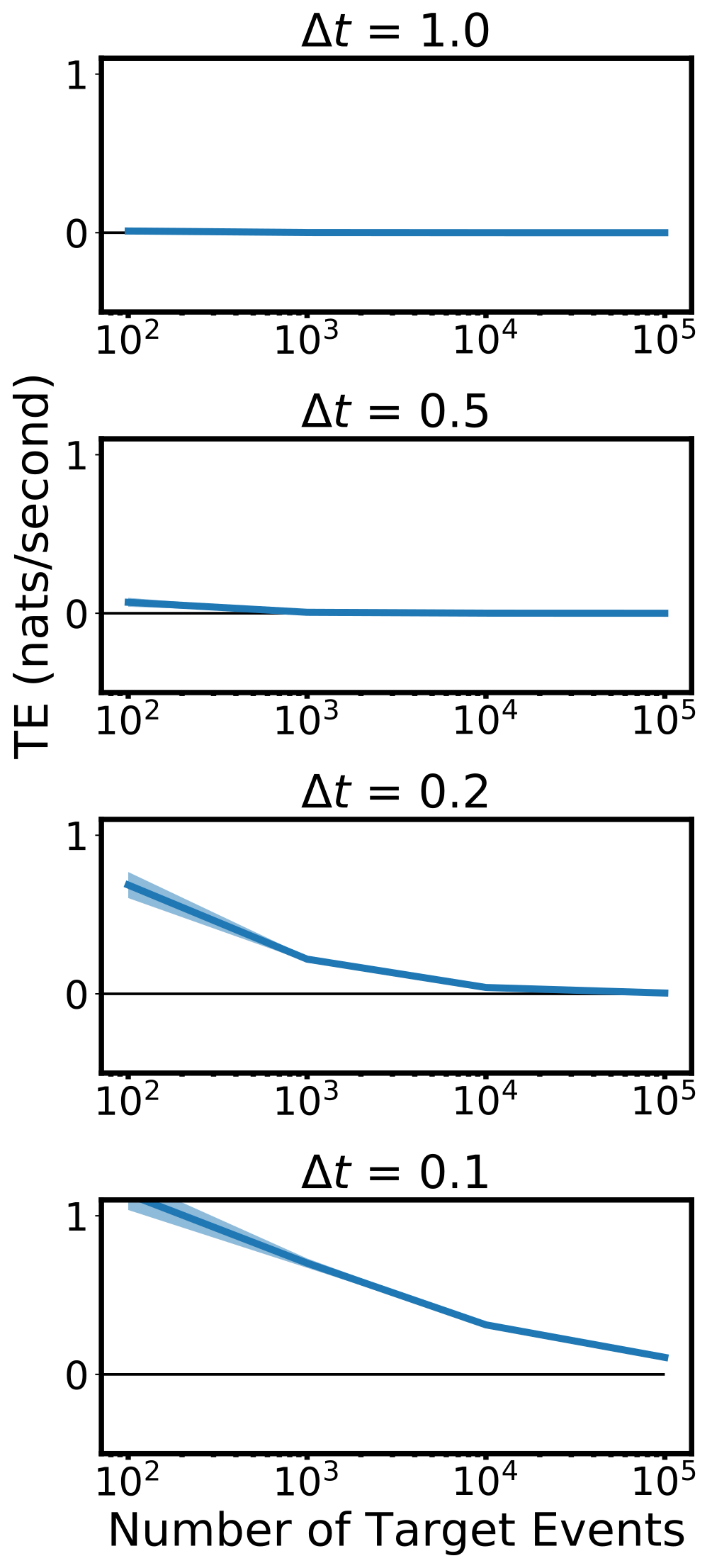

Supplement: S2 Fig — The results of an identical experimental setup to those displayed in Fig 3, but where the history embedding lengths (l and m) were set to cover the distance of an average interspike interval. Specifically, these lengths were 1, 2, 5 and 10, corresponding to the Δt values of 1.0, 0.5, 0.2 and 0.1. (TIFF) [file pcbi.1008054.s002.tiff]

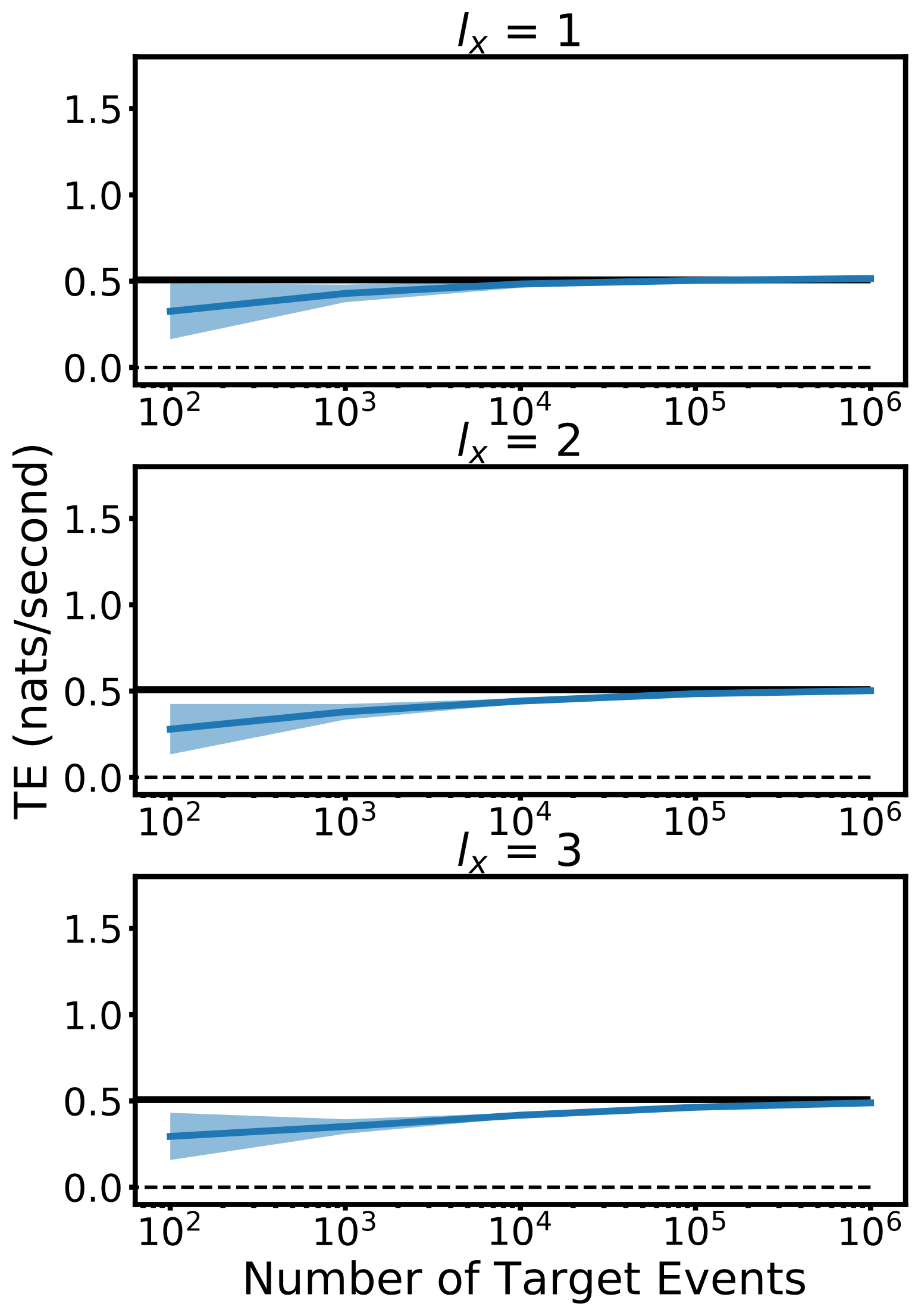

Supplement: S3 Fig — The results of an identical experimental setup to those displayed in Fig 4B, but where the history embedding length of the source is set to lY = 3. (TIFF) [file pcbi.1008054.s003.tiff]

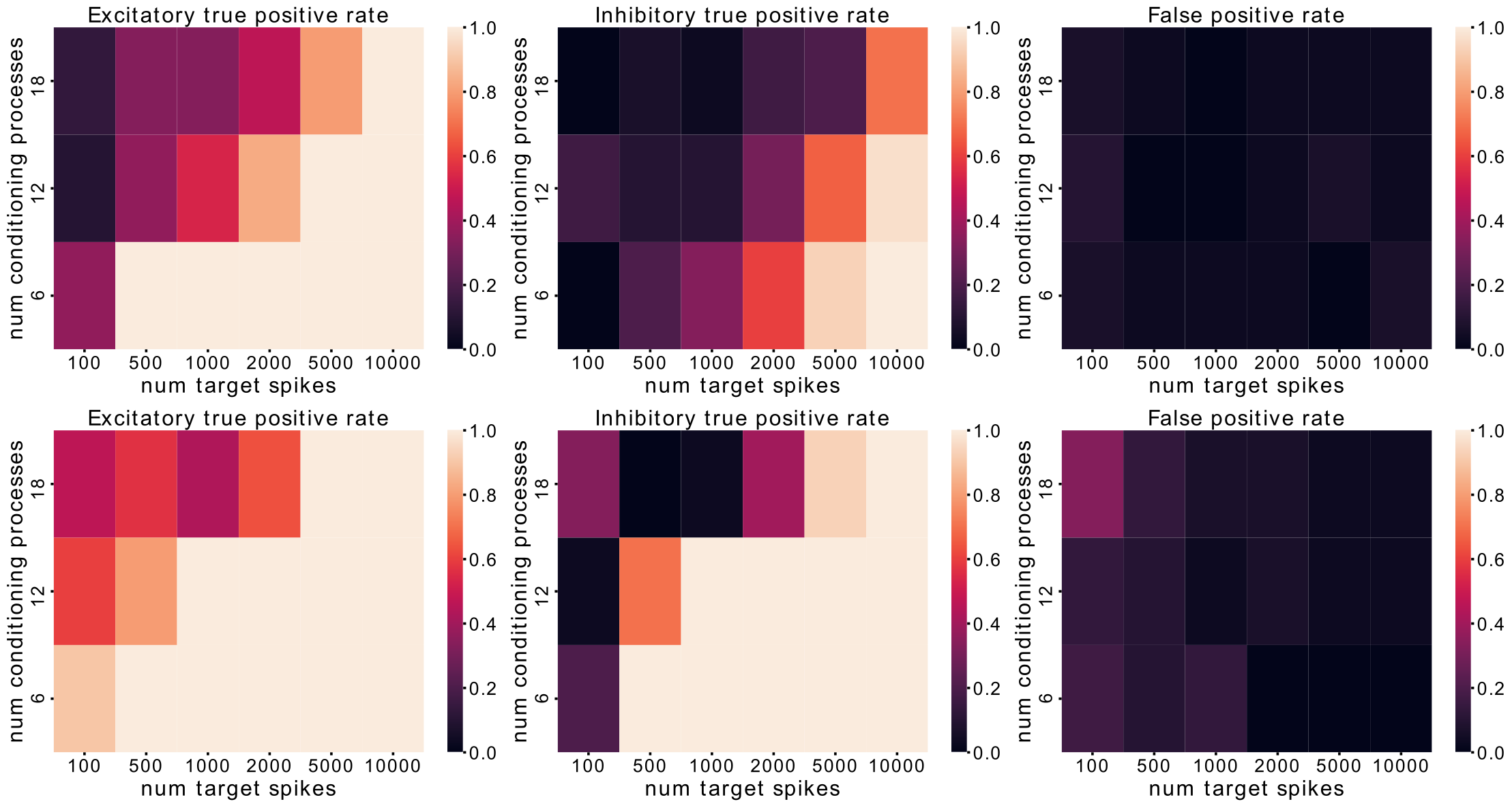

Supplement: S4 Fig — The results of an identical experimental setup to those displayed in Fig 8, but with a constant rate of 20 Hz in all the stimuli. This removes the correlation between the unconnected source and the firing of the target. The top row shows results of the continuous-time approach, the bottom shows results of the discrete-time approach. (TIFF) [file pcbi.1008054.s004.tiff]

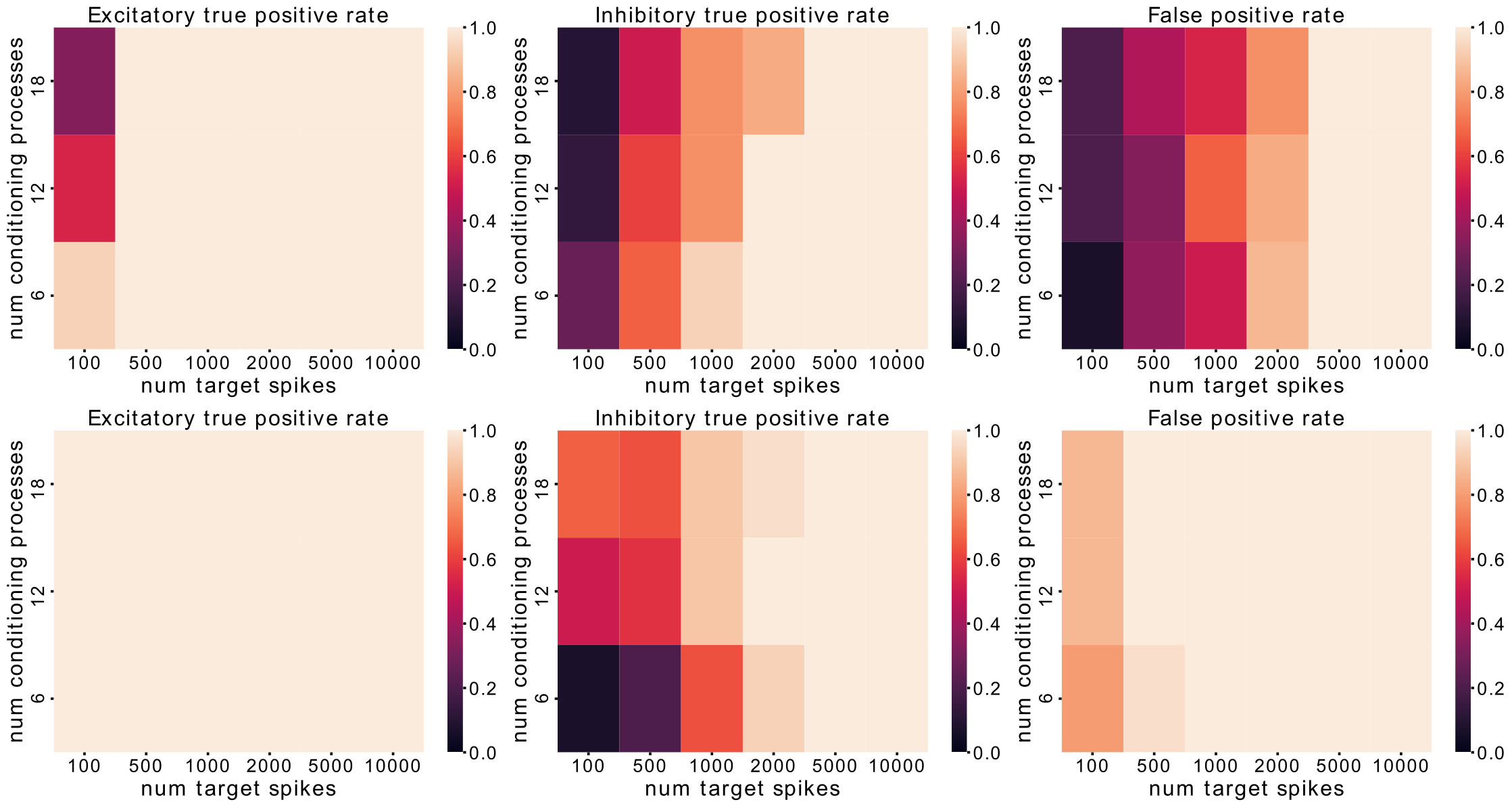

Supplement: S5 Fig — The results of an identical experimental setup to those displayed in Fig 8, but where the background processes are not included in the conditioning set (the conditioning set is left empty). This represents the nature of the inference task at the early stage of a greedy network inference algorithm being applied to a node. We see that the continuous-time estimator performs well on inhibitory connections in this case. Due to the change in dimension, different source and target embedding lengths (l and m) as well as bin widths Δt were used for the discrete-time estimator. These were set at l = m = 12 and Δt = 2ms. The top row shows results of the continuous-time approach, the bottom shows results of the discrete-time approach. (TIFF) [file pcbi.1008054.s005.tiff]

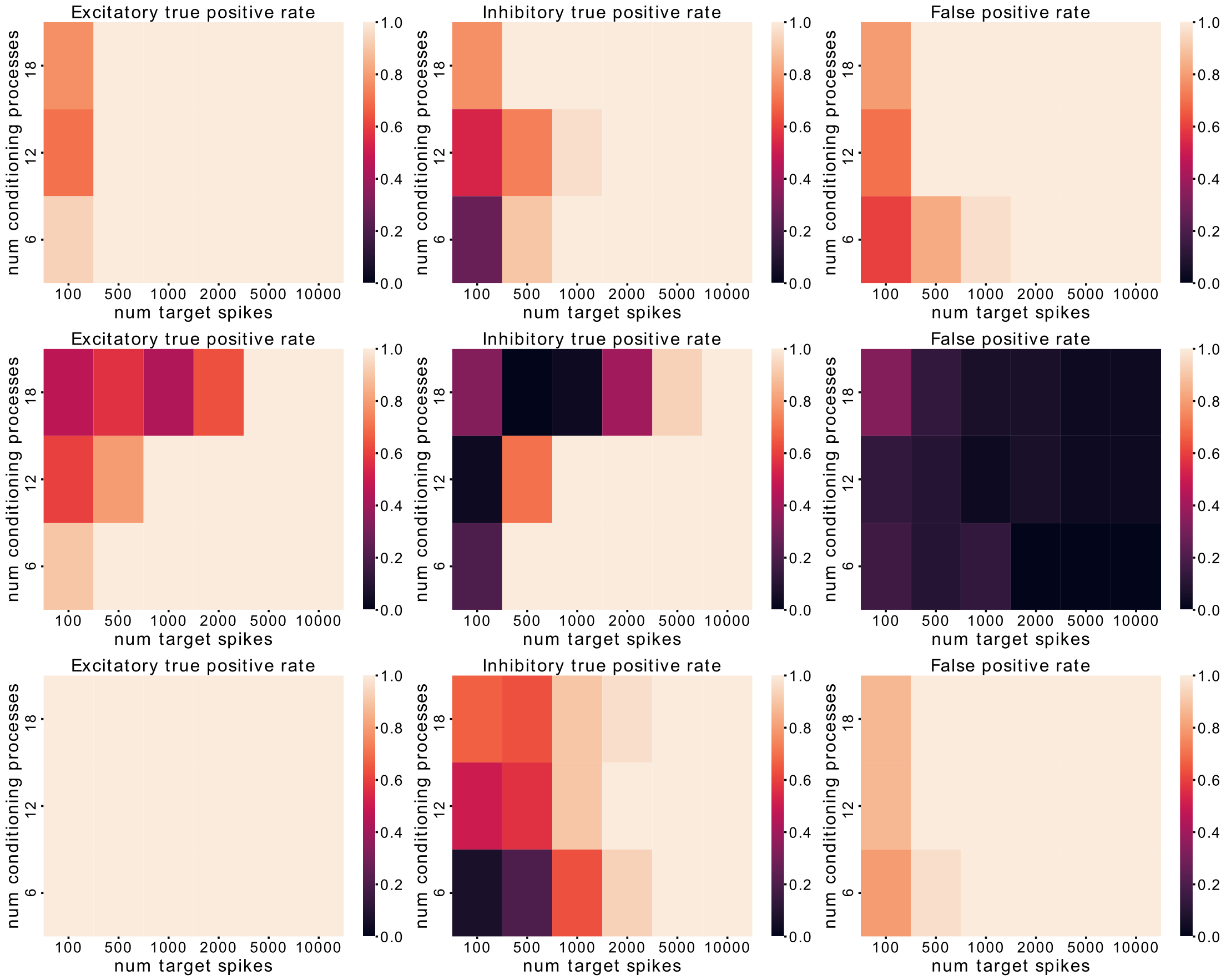

Supplement: S6 Fig — The results of identical experimental setups to those displayed in the bottom rows of Fig 8, S4 and S5 Figs. As the bottom rows of all of these figures show the results of the discrete-time estimator, the plots in this figure similarly all display the results of runs of the discrete-time estimator. However, where the other plots make use of the source time-shift method for surrogate generation (as is traditionally used in conjunction with TE estimators), these plots make use of a standard conditional-permutation-based surrogate generation scheme for categorical variables [64]. The top row of this figure corresponds to the bottom row of Fig 8, the middle row corresponds to the middle row of S4 Fig and the bottom row corresponds to the bottom row of S5 Fig. (TIFF) [file pcbi.1008054.s006.tiff]

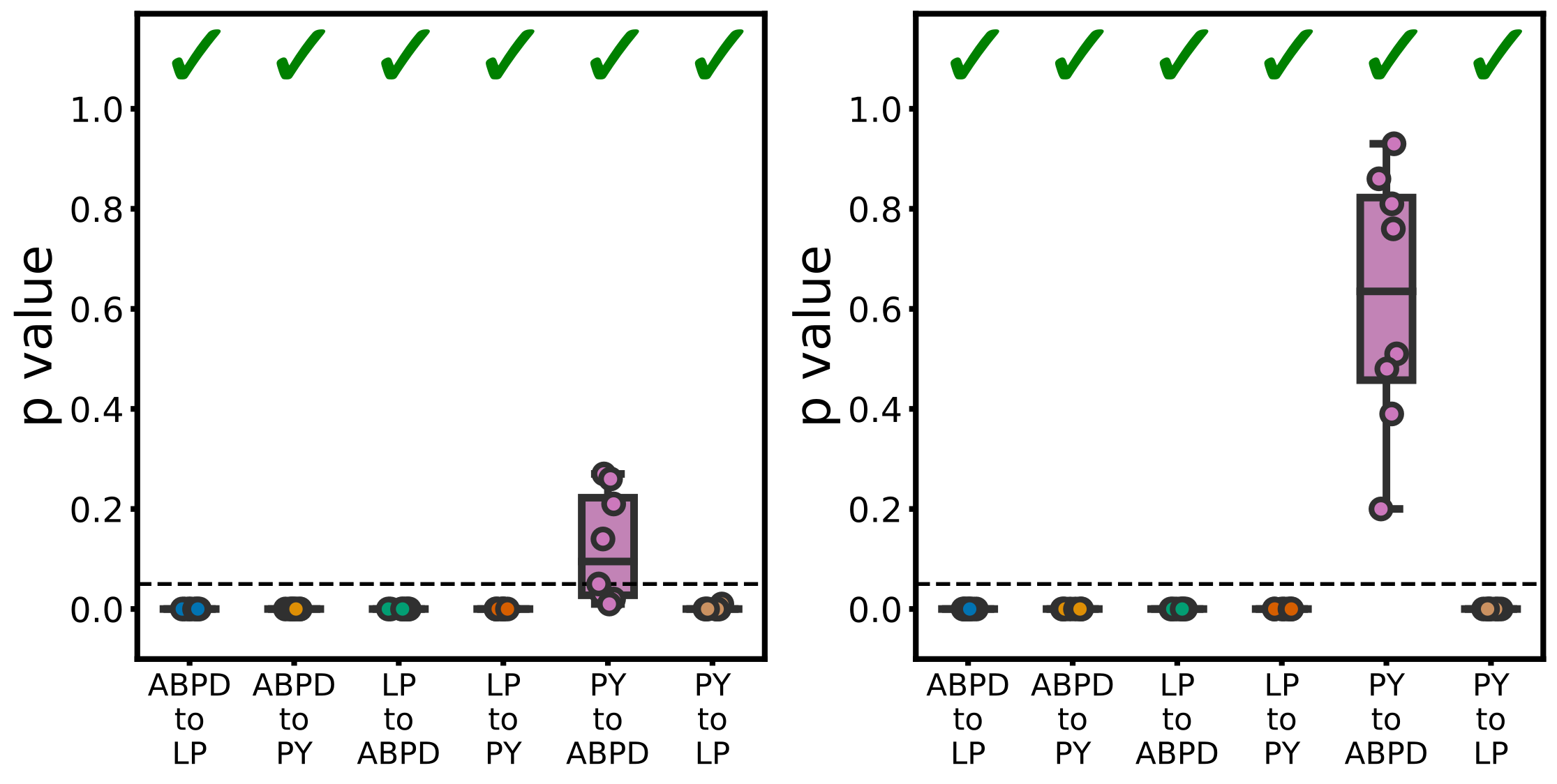

Supplement: S7 Fig — The results of an identical experimental setup to those displayed in Fig 9C, but where different embeddings lengths (lX, lY and lZ1) are used. The left plot shows lX=lY=lZ1=2 and the right plot shows lX=lY=lZ1=4. (TIFF) [file pcbi.1008054.s007.tiff]

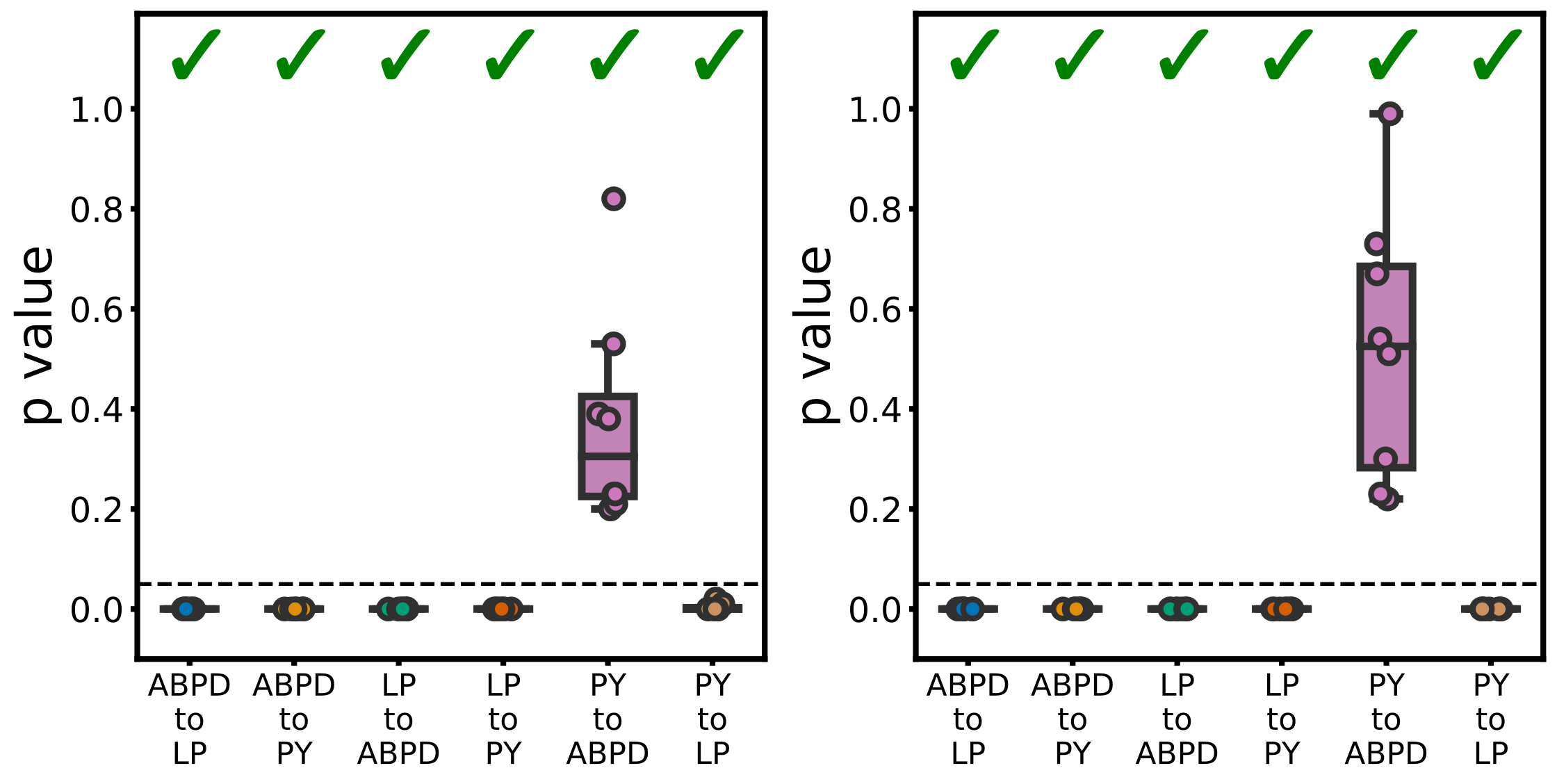

Supplement: S8 Fig — The results of an identical experimental setup to those displayed in Fig 9C, but where different numbers of target spikes NX are used. The left plot shows NX = 1 × 4 and the right plot shows NX = 3.5 × 4. (TIFF) [file pcbi.1008054.s008.tiff]
