# Supplementary material for: Estimating Transfer Entropy in Continuous Time Between Neural Spike Trains or Other Event-Based Data
Source: PLoS Comput Biol. 2021 Apr 19;17(4):e1008054. doi: 10.1371/journal.pcbi.1008054 (PMC8084348; doi:10.1371/journal.pcbi.1008054)
Supplement: S1 Text — (PDF) [file pcbi.1008054.s009.pdf]

Supplementary Text S9 for Estimating Transfer Entropy in Continuous Time Between Neural Spike Trains or Other Event-Based Data

David P. Shorten, Richard E. Spinney and Joseph T. Lizier

Specification of Biophysical Network Models Inspired by the Pyloric Circuit of the Crustacean Stomatogastric Ganglion

Current	E	p	q	m_∞	h_∞
I_{Na}	50	3	1	$\frac{1}{1+\exp\left(\frac{V+25.5}{-5.29}\right)}$	$\frac{1}{1+\exp\left(\frac{V+48.9}{5.18}\right)}$
I_{CaT}		3	1	$\frac{1}{1+\exp\left(\frac{V+27.1}{-7.2}\right)}$	$\frac{1}{1+\exp\left(\frac{V+32.1}{5.5}\right)}$
I_{CaS}		3	1	$\frac{1}{1+\exp\left(\frac{V+33}{-8.1}\right)}$	$\frac{1}{1+\exp\left(\frac{V+60}{6.2}\right)}$
I_A	-80	3	1	$\frac{1}{1+\exp\left(\frac{V+27.2}{-8.7}\right)}$	$\frac{1}{1+\exp\left(\frac{V+56.9}{4.9}\right)}$
I_{KCa}	-80	4	0	$\frac{[\mathrm{Ca}]}{[\mathrm{Ca}]+3}\frac{1}{1+\exp\left(\frac{V+28.3}{-12.6}\right)}$	
I_{Kd}	-80	4	0	$\frac{1}{1+\exp\left(\frac{V+12.3}{-11.8}\right)}$	
I_H	-20	1	0	$\frac{1}{1+\exp\left(\frac{V+75}{5.5}\right)}$	

Table 1: Parameters and functions used in the conductance based model.

We devised a simulation approach which follows very closely that presented in [1, 2, 3]. The only significant deviation is the addition of a noise term.

We modelled the neurons of the pyloric circuit using a conductance-based model. The membrane potential (V) evolves according to

$$C\frac{dV}{dt} = -\sum_i I_i - \sum_s I_s + \xi. \tag{1}$$

$C = 0.628\,\mathrm{nF}$ is the membrane conductance. ξ is a noise term. Each current is specified by

$$I_i = g_i m_i^{q_i} h_i^{p_i}(V - E_i).$$

E_i is the reversal potential and its values are listed in table 1. The reversal potentials associated with the calcium channels are not listed as these are calculated according to the

Current	τ_m	τ_h
I_{Na}	$2.64 - \dfrac{2.52}{1+\exp\left(\frac{V+120}{-25}\right)}$	$\dfrac{1.34}{1+\exp\left(\frac{V+62.9}{-10}\right)}\left[1.5 - \dfrac{42.6}{1+\exp\left(\frac{V+34.9}{3.6}\right)}\right]$
I_{CaT}	$43.4 - \dfrac{42.6}{1+\exp\left(\frac{V+68.1}{-20.5}\right)}$	$210 - \dfrac{179.6}{1+\exp\left(\frac{V+55}{-16.9}\right)}$
I_{CaS}	$2.8 + \dfrac{14}{\exp\left(\frac{V+27}{10}\right)+\exp\left(\frac{V+70}{-13}\right)}$	$120 + \dfrac{300}{\exp\left(\frac{V+55}{9}\right)+\exp\left(\frac{V+65}{-16}\right)}$
I_A	$23.2 - \dfrac{20.8}{1+\exp\left(\frac{V+32.9}{-15.2}\right)}$	$77.2 - \dfrac{58.4}{1+\exp\left(\frac{V+38.9}{-26.5}\right)}$
I_{KCa}		$180.6 - \dfrac{150.2}{1+\exp\left(\frac{V+46}{-22.7}\right)}$
I_{Kd}		$14.4 - \dfrac{12.8}{1+\exp\left(\frac{V+28.3}{-19.2}\right)}$
I_H		$\dfrac{2}{\exp\left(\frac{V+169.7}{-11.6}\right)+\exp\left(\frac{V-26.7}{14.3}\right)}$

Table 2: Parameters and functions used in the conductance based model.

Nernst equation. Specifically, $E_{\mathrm{Ca}} = \frac{RT}{2F}\log_{10}\left(\frac{[\mathrm{Ca}^{2+}]_{\mathrm{ext}}}{[\mathrm{Ca}^{2+}]}\right)$ where $R = 8.314\,\mathrm{J\,K^{-1}\,mol^{-1}}$ is the universal gas constant, $T = 293.3\,\mathrm{K}$ is the temperature and $F = 96\,485.332\,12\,\mathrm{C\,mol^{-1}}$ is Faraday's constant. $[\mathrm{Ca}^{2+}]_{\mathrm{ext}} = 3\,\mathrm{mM}$ is the extracellular Ca^{2+} concentration and $[\mathrm{Ca}^{2+}]$ is the intracellular Ca^{2+} concentration. The intracellular Ca^{2+} concentration evolves according to

$$\tau_{\mathrm{Ca}}\frac{d[\mathrm{Ca}^{2+}]}{dt} = -f\left(I_{\mathrm{CaT}} + I_{\mathrm{CaS}}\right) - [\mathrm{Ca}^{2+}] + [\mathrm{Ca}^{2+}]_0.$$

$[\mathrm{Ca}^{2+}]_0 = 0.05\,\mathrm{\mu M}$ is the steady-state Ca^{2+} concentration, $f = 14.96\,\mathrm{\mu M\,nA^{-1}}$ and $\tau_{\mathrm{Ca}} = 200\,\mathrm{ms}$

The values of q_i and p_i are listed in table 1. The activation variables m_i evolve according to

$$\tau_{m_i}\frac{dm_i}{dt} = m_{\infty,i} - m_i.$$

The inactivation variables h_i evolve according to

$$\tau_{h_i}\frac{dh_i}{dt} = h_{\infty,i} - h_i.$$

$m_{\infty,i}$ and $h_{\infty,i}$ are given in table 1 and τ_{m_i} and τ_{h_i} are given in table 2.

The synaptic currents are specified by

$$I_s = g_s a(V - E_s).$$

E_s is the synaptic reversal potential. It was set to $-70\,\mathrm{mV}$ for glutamatergic synapses and $-80\,\mathrm{mV}$ for cholinergic synapses.

The activation variable a_s evolves according to

$$\tau_{a_s}\frac{da_s}{dt} = a_{\infty,s} - a_s$$

where

$$\tau_{a_s} = \frac{1-a_s}{k_-}.$$

In the previous work which we are following [1, 2, 3], $a_{\infty,s}$ was a function of the presynaptic membrane potential. The functional form was such that the majority of current flow across the synapse was in the vicinity of spikes. However, the fact that all the influence over the synapse is not contained in the spikes opens the possibility for causal sufficiency not being met. As the principal purpose of this model in the context of this paper is to produce examples in which we have a known ground-truth of conditional dependence/independence we made a slight modification to the model to ensure that this condition was met. We specified that $a_{\infty,s}$ would evolve according to

$$\tau_{a_{\infty,s}}\frac{da_{\infty,s}}{dt} = 0 - a_{\infty,s}$$

where $\tau_{a_{\infty,s}} = 25\,\mathrm{ms}$. On the occurrence of a presynaptic spike $a_{\infty,s}$ was set to 0.99.

$\Delta = 5\,\mathrm{mV}$ provides the slope of the activation curve. $V_{\mathrm{th}} = -35\,\mathrm{mV}$ is the half activation potential of the synapse. V_{pre} is the membrane potential of the presynaptic neuron. k_- is the rate constant for the transmitter-receptor dissociation rate. For the glutamatergic synapses we used $k_- = 0.025\,\mathrm{ms}$ and for the cholinergic synapses we used $k_- = 0.01\,\mathrm{ms}$.

Simulations of the pyloric rhythm can be run with fixed maximum conductance values g_i and g_s, as in [2]. However, it was found that these models were less robust to the addition of a noise term. It was, therefore, decided to use adaptive conductances as described in [3]. Each conductance evolved according to:

$$\tau_g\frac{dg_j}{dt} = m_j - g_j$$

where

$$\tau_{m_j}\frac{dm_j}{dt} = [\mathrm{Ca}^{2+}] - \mathrm{Ca}_{\mathrm{tgt}}.$$

$\tau_g = 100\,\mathrm{ms}$ was common across all channels. The time constants τ_{m_j} are listed in table 3. The time constants provided in [3] were not used as these produce a pyloric rhythm with unrealistically short period. Instead, the approach presented in [3] for arriving at conductance time constants from desired conductance values was used. The conductance values in [2] were used as these desired values. Specifically, we used the values presented in table 2 in [2] for AB/PD 1, LP 2 and PY 1. The time constant τ_{m_j} associated with g_j was set as $\tau_{m_j} = c/g_j$. c is a constant with units of seconds that was adjusted by hand so that the activity converged in a reasonable amount of time and the time constants were of the same order of magnitude as those provided in [3]. As in [3], the leak conductances were fixed. They were set at 0 for the AB/PD neuron, and $0.0628\,\mu\mathrm{S}$ for the PY neuron and $0.1256\,\mu\mathrm{S}$ for the LP neuron.

Conductance	AB/PD	LP	PY
g_{Na}	0.25	1	1
g_{CaT}	40	1e15	40
g_{CaS}	16.67	25	40
g_{A}	2	5	2
g_{KCa}	10	20	1e15
g_{Kd}	1	4	0.8
g_{H}	1×10^4	2×10^3	2×10^3

Table 3: The conductance time constants τ_{m_j}. All values are in seconds.

LP $\rightarrow$ AB/PD, glutamatergic	2×10^4
AB/PD $\rightarrow$ LP, cholinergic	500
AB/PD $\rightarrow$ LP, glutamatergic	1×10^4
PY $\rightarrow$ LP, glutamatergic	1×10^4
AB/PD $\rightarrow$ PY, cholinergic	5×10^3
AB/PD $\rightarrow$ PY, glutamatergic	250
LP $\rightarrow$ PY, glutamatergic	1×10^6

Table 4: The conductance time constants τ_{m_j} for the synapses. All values are in seconds.

The TE approach to network inference will not work in a fully deterministic system. As such, noise was added to the system. There are a number of techniques for adding noise to conductance-based models [4]. The simplest such technique is to add noise to the currents in (1). Although this is not a biophysically realistic method, it has been shown to produce resulting behaviours which closely match those produced by more realistic techniques [4, 5]. As such, we decided to make use of this procedure in our simulations. The associated noise term is shown in (1). The noise was generated using an AR(1) process

$$\xi_t = \theta \xi_{t-1} + \epsilon_t. \tag{2}$$

We used the parameter value of $\theta = 0.005$. ϵ_t was distributed normally with mean 0 and standard deviation 9×10^{-9}

A simulation timestep of $\Delta t = 0.005\,\text{ms}$ was used.

References

[1] Prinz AA, Billimoria CP, Marder E. Alternative to hand-tuning conductance-based models: construction and analysis of databases of model neurons. Journal of Neurophysiology. 2003;90(6):3998–4015.

[2] Prinz AA, Bucher D, Marder E. Similar network activity from disparate circuit parameters. Nature Neuroscience. 2004;7(12):1345.

[3] O'Leary T, Williams AH, Franci A, Marder E. Cell types, network homeostasis, and pathological compensation from a biologically plausible ion channel expression model. Neuron. 2014;82(4):809–821.

[4] Goldwyn JH, Shea-Brown E. The what and where of adding channel noise to the Hodgkin-Huxley equations. PLoS Computational Biology. 2011;7(11).

[5] Rowat P. Interspike interval statistics in the stochastic Hodgkin-Huxley model: Coexistence of gamma frequency bursts and highly irregular firing. Neural Computation. 2007;19(5):1215–1250.
