# Supplementary material for: Estimating Transfer Entropy in Continuous Time Between Neural Spike Trains or Other Event-Based Data
Source: PLoS Comput Biol. 2021 Apr 19;17(4):e1008054. doi: 10.1371/journal.pcbi.1008054 (PMC8084348; doi:10.1371/journal.pcbi.1008054)
Supplement: Attachment — Submitted filename: Response.pdf [file pcbi.1008054.s010.pdf]

21 September 2020

Article: *Estimating Transfer Entropy in Continuous Time Between Neural Spike Trains or Other Event-Based Data*

Authors: David P. Shorten, Richard E. Spinney, Joseph T. Lizier

Dear Editors and Reviewers, We thank you for the constructive feedback and comments. We now submit a revised version of the manuscript in light of these comments, and a list of responses to them. We feel that these revisions have made a positive contribution to the manuscript.

We present a general response to a broader issue which affected multiple reviews below. We subsequently address each review individually. In the review-specific section, the comments of reviewers are shown in black, our responses are shown in blue, and specific descriptions of changes to the text are described in magenta.

An issue which was common across most of the reviews was the interpretation that this paper was primarily about the inference of underlying structural (causal) networks. This was not intended to be the case; to be specific, the intended focus of the paper is as follows:

1. The derivation of an estimator of Transfer Entropy (TE) for spike trains.
2. Demonstration that this estimator is superior to the previous approach of time binning by:
   (a) being a consistent estimator (that is, it converges to the true value of the TE).
   (b) having lower bias.
   (c) converging faster as we increase available data.
3. The derivation of a surrogate generation scheme (consistent with conditional independence of source and target transitions) which improves on previous approaches by correctly maintaining correlations between the history of the source and the histories of the other processes.
4. Making the point that the combination of the proposed estimator and surrogate generation scheme is a test for conditional independence.
   (a) Demonstrating that this test has high accuracy on a number of challenging synthetic examples on which the traditional TE approach fails (the combination of time binning and surrogates created by shifting the source in time).
   (b) In order to validate this test on more biologically faithful examples, we instantiated a simulation of a biophysical model of a small neural circuit. This model was specifically chosen because it met certain conditions which allowed us to know the ground truth of the conditional dependence/independence relationships between nodes (which has a one-to-one correspondence with structural connectivity here). This then provided us with an additional validation of our conditional independence test with a known ground truth.

We do foresee that one of the more useful applications of the conditional independence test that the combination of estimator and surrogate generation scheme provides will be network inference. The importance of being able to accurately infer the connectivity of spiking neural networks under the assumption of full observability was highlighted in a very recent Nature Neuroscience paper [6]. Here,

it was shown that popular existing approaches to this problem, such as generalised linear models and maximum entropy-based reverse Ising inference, had very high false-positive rates in instances where the activity of unconnected neurons was highly correlated. Given our focus on demonstrating that our conditional independence test is highly robust to strong pairwise correlations despite conditional independence, we believe that the work presented in this paper holds great promise towards making progress on this issue. That being said, any conditional independence test will suffer from the curse of dimensionality. This means that performing effective network inference on larger networks requires pairing the conditional independence test with a suitable (conditional-independence-based) network inference algorithm which reduces the dimensionality of the tests. A variety of such algorithms exist [14, 7]. This paper does not demonstrate pairing this conditional independence test with one of those algorithms. As such, it does not aim to speak to network inference. This is a topic which is left to future work.

Whilst we are interested in the efficacy of the pairing of the conditional independence test with a network inference algorithm in future, we believe this is out of scope for this article for the following reasons:

1. As any conditional independence test can be used in conjunction with multiple different network inference algorithms, there is an advantage to publishing the test separately.
2. The various conditional-independence-based network inference algorithms are well established and have been thoroughly studied. Their scaling is governed by the scaling of the conditional independence test, which we now demonstrate in our revised manuscript.
3. There is precedent for publishing conditional independence tests in isolation without any associated demonstration of network inference. They have been published in many well-regarded journals and conference proceedings [1]
4. We are not only presenting a conditional independence test, but the first consistent estimator for a quantity of interest when studying spike trains (outside of the context of network inference).
5. The article is very long already.

In our experiments using data from a simulation of the pyloric network of the crustacean stomatogastric ganglion, as the network is only composed of three nodes, the few low-dimensional conditional-independence tests that we perform are sufficient to infer the network. However, we appreciate that using the term "connectivity inference" and other related language in the context of this specific example was misleading regarding our goal. We realise that many readers would assume from this that we are presenting a network inference algorithm. As such, all such language relating to these experiments has been removed. Instead, we now emphasise that this network is used to provide known independence/dependence relationships on which we can test our algorithm. These changes are listed below, before we address each individual review separately.

We thank you again for considering our manuscript, and look forward to your response.

David Shorten, for and on behalf of the authors.

[1] These include *NeurIPS* [20, 4, 11], *AISTATS* [19], *Frontiers in Psychology*[9], *Econometric Theory* [24, 23], and the *Review of Economics and Statistics*[1]

Updated text 0.1:

- The last sentence of the abstract has been changed from

  > The power of this approach is further demonstrated on the inference of the connectivity of biophysical models of a spiking neural circuit inspired by the pyloric circuit of the crustacean stomatogastric ganglion

  to

  > This capacity to accurately test for conditional independence is further demonstrated on models of a spiking neural circuit inspired by the pyloric circuit of the crustacean stomatogastric ganglion.

- On lines 22 and 23, "our correct inference of the underlying connection structure" has changed to "our correct inference of these flows".
- The previous subsection D of the results (now subsection E) has been substantially reworded.
- The paragraph of the discussion describing this subsection has been reworded to:

  > Finally, our proposed approach was applied to inferring the dependence/independence relationships in a more biologically faithful example in Results Sec. II E. For this purpose, we made use of models of the pyloric circuit of the crustacean stomatogastric ganglion. The full observability and large noise provided by this model allowed us to conclude that the conditional dependence/independence relationships would match the underlying connectivity of the model, thus providing us with a ground truth against which to test our approach. Statistical modelling of this network is challenging due to its highly periodic dynamics. For instance, attempts to use Granger causality, using a more established estimator, to infer its connectivity have been unsuccessful [64]; furthermore, we showed that the discrete-time binary-valued TE estimator (with time-shifted surrogates) also could not successfully infer the independence and dependence relationships in the network. It is worth highlighting in this context that TE is a non-linear version of Granger causality [66]. This makes us suspect that the reason for the earlier failures of Granger causality applied to this network was due, at least in part, to time binning and not entirely due to its inability to find non linear relationships. Despite these challenges, our combination of continuous-time estimator and surrogate generation scheme was able to correctly infer the relationships implied by the pyloric network. This provides an important validation of the efficacy of our presented approach on a challenging example of representative biological spiking data.

- The end of the introduction now reads

  > Our results provide strong impetus for the application of our proposed techniques to investigate information flows in spike-train data recorded from biological neurons. Further186 more, we underline the importance of our correct identification of conditional dependence and independence in these experiments. Whilst functional/effective network inference algo188 rithms using TE estimators such as ours are not expected to align with structural networks in general, they would be expected to do so under certain idealised assumptions (e.g. full observability, large sample size, etc., as outlined in Results part C) implemented in these experiments. As recently discussed by Novelli and Lizier [47], and specifically for spiking neural networks by Das and Fiete [48], inference aligning with underlying structure under such conditions is a crucial validation that the effective network models they infer at scale are readily interpretable. As such,

the demonstration of the efficacy of our proposed approach in small networks here implies that it holds promise for larger scale effective network inference once paired with a suitable (conditional-independence-based) network inference algorithm (e.g. IDTxl as described in [7, 49])

- A new paragraph has been added to the end of the discussion further explaining how this work fits into the wider context of network inference.

1 Reviewer 1

This paper introduces a method and an empirical approach for estimating transfer entropy for event-based data. It avoids the time-descritization by operating on the probability densities of the history embeddings of the variables, rather than making a direct estimation of spike rates. Authors also provide an adaptation of a recently proposed surrogate generation scheme in order to have a complete statistical test to provide statistical strength of the conditional independence under consideration.

Major comments

Overall the paper is well written. However, special care should be taken in the causal claims and statements that authors make. How will the proposed method perform in the case that $X_{t-1} \rightarrow Y_t$ and $Y_{t-1} \rightarrow X_t$ and $X_{t-s} \not\rightarrow X_t$ and $Y_{t-s} \not\rightarrow Y_t$, for all $s > 0$, $t > 0$? According to Janzing et al. 2013 (see section 5.2 in Quantifying causal influences, The Annals of Statistics 41 (5), 2324-2358.), transfer entropy will always converge to 0 in this particular example although there is causal influence between X and Y. That means that there are cases in which TE will not give correct information. Therefore, authors should mild the sentences were they connect TE estimation to causal statements and they should also discuss that their TE estimator and surrogate scheme assumes acyclic summary graphs and causal sufficiency.

Response 1.1: As per the comments to all reviewers above, this paper is not about causal inference, although we do use underlying causal models to establish conditional dependence and independence as tests for our estimator.

Along these lines, we do not make the claim that non-zero TE implies a causal connection in general. Author JL has previously described why the concept of modelling information transfer (via transfer entropy) and causality are distinct, whilst both are useful [10]. Specifically, the differences in TE and causality for the example highlighted by the reviewer – first presented by Ay and Polani, 2008 [2] – were interpreted in that paper, and also JL's 2016 TE book [5] (see section 4.2.2.1 where a similar example is considered). TE simply provides a test of conditional independence, which can be interpreted as a model of information transfer, which is distinct from causality.

This test for conditional independence can be used as a component in a network inference algorithm. The degree to which such an inferred network matches the underlying causal structure depends on various conditions and assumptions being met. There is a large literature on what can be inferred about causal relationships from such a test and its combination with network inference algorithms. However, covering this literature in depth is beyond the scope of this paper, which is already very long.

What the reviewer's comments have highlighted for us is that, in some of the test cases we used, determining the ground-truth of independence vs dependence against which we compare our inferred values does require making use of various assumptions (such as faithfulness, sufficiency and the causal Markov property) which we did not make clear. These assumptions need to be made more explicit. We thank the reviewer for bringing this to our attention.

We also appreciate that, in some places, our use of language left room for misinterpretation. In particular, we realise that our use of the phrase "connectivity inference" (and related language) could be

misleading to some readers. We appreciate the reviewer highlighting this lack of clarity in our writing. We have made modifications as described in our comments to all reviewer above, and specifically to this reviewers comments as follows:

Updated text 1.1:

1. Abstract: "signifying the lack of a causal connection under certain weak assumptions." has been removed.
2. Line 48:

   > Such conditional independence implies the absence of an active causal connection between X and Y under certain weak assumptions, and TE is widely used for inferring directed functional and effective network models

   has been changed to

   > Such a conditional independence test can be used as a component in a network inference algorithm and, as such, TE is widely used for inferring directed functional and effective network models
3. A new subsection D has been added to the methods. This subsection makes explicit the assumptions that we use to conclude the ground-truth of conditional dependence/independence in the models on which we test our approach.
4. Language referring to "connectivity" has been updated to highlight that, at this stage, we are simply presenting a test for conditional independence (along with the first consistent estimator of TE on spike trains). Network inference will follow in subsequent work. Please refer to the cover letter and Updated Text 0.1 for details.

Line 46: Authors should define the meaning of "active" causal connection between X and Y. Do you imply a connection that does not include any hidden variable? If that is what the authors want to say they need to mention they assume causal sufficiency.

Response 1.2: Please see response 1.1 and Updated Text 1.1 (point 2 in particular).

Line 49: Authors should particularly present the assumptions they imply here. Do they mean the necessary assumption for relating conditional independence tests and d-separation statements in causal graphs? ("Causal Faithfulness" and "Causal Markov Condition").

Response 1.3: Please see response 1.1 and Updated Text 1.1.

Line 179: Authors claim that non-zero TE values imply a directed relationship. That is not true if Causal Sufficiency (all common causes are observed) is not assumed. In presence of hidden confounders $Y \leftarrow H \rightarrow X$ the TE for Y and X will be different from zero, as H cannot be observed. Nevertheless this is not a directed relationship between Y and X. Authors need to write that this is true only under causal sufficiency.

Response 1.4: The sentence in question is "The comparison of the estimates to a population of surrogates produces p-values for the statistical significance of the TE values being distinct from zero and implying a directed relationship." This sentence does not imply causality. Causal relationships are not the only type of relationship. This sentence is simply reflecting the fact that TE implies that the present of the target is statistically dependent on the history of the source. That being said, we now realise the possibility of this sentence being misinterpreted.

Updated text 1.2: The sentence has been changed from

> The comparison of the estimates to a population of surrogates produces p-values for the statistical significance of the TE values being distinct from zero and implying a directed relationship.

to

> The comparison of the estimates to a population of surrogates produces p-values for the statistical significance of the TE values being distinct from zero and implying a directed statistical dependence.

Figure 2: The figure writes Ns/Nx while the caption and in the text writes Nu/Nx. Please be consistent with your notation across the main text and the figures.

Response 1.5: This has been fixed. Thanks for spotting this typo.

Line 208: The authors write "the averages are taken across 1000, 100, 100 and 100 tested process pairs respectively." Why the first one is averaged over 1000 and the other three over 100?

Response 1.6: For lower numbers of target spikes, the estimator has a higher variance. We therefore want to average over more runs in order to get a better estimate of the mean. For longer sequences, the computational cost increases, and so running over many examples becomes infeasible. However, the much lower variance on these longer sequences means that we can still get a good estimate of the mean. In order to provide an example of the lack of sensitivity of our results to these choices, we reran the experiment for figure 2. The plots in the text are constructed from averages taken over 1000, 100, 20 and 20 examples. We increased this to 1000, 100, 100 and 100 examples. The plots with this increased number of samples is shown below. They are visually identical to those in figure 2.

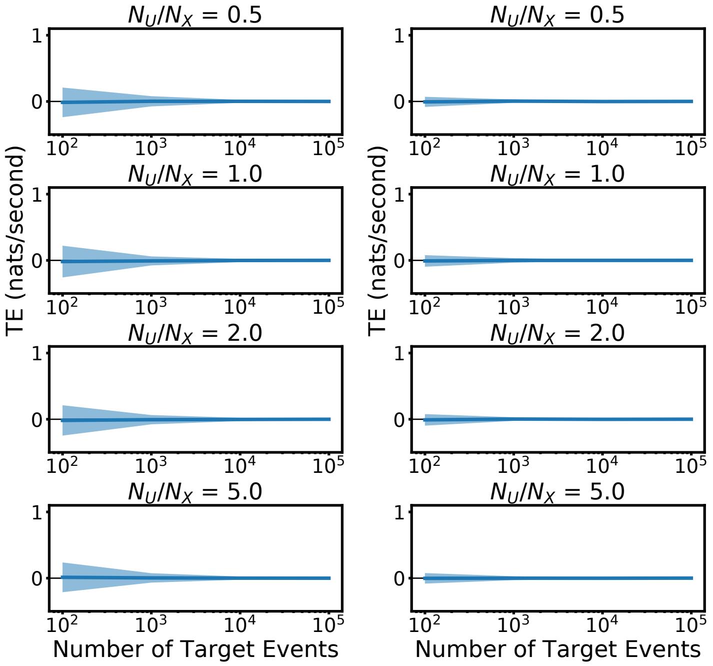

Line 262: Causal statements should be made with cautiousness. Authors need to specify the assumptions they imply. Causal faithfulness and Causal Markov Condition (if these are the implied ones) are not weak assumptions. In addition causal sufficiency which is necessary for granger causality is a very strict and hard to meet assumption. Its violation renders TE inadequate for causal discovery.

Response 1.7: Please see response 1.1 and Updated Text 1.1. In particular, the added paragraph listed there is included in the paper just below line 262.

Line 491: Regarding the successful application of the method on the pyloric network of the crustacean stomatogastric ganglion: I have some questions. First of all, the referenced paper that used Granger Causality, used a linear implementation. Does the preprocessing of the data differ between the two papers? Did the authors compared directly on their data with GC? If so, did they try both linear and non linear implementations of GC? Since both GC and the authors' method suffers from hidden confounding both would be expected to fail in a real scenario with hidden confounders. Were all the necessary information observed in the presented experiment? If yes, then it should be stressed accordingly because this is an ideal scenario. Could the authors elaborate on their intuition why their method worked successfully compared to Granger? Is it because their method is specifically designed for event-based data? Could the linear implementation be the reason of the many false positives of Granger method?

Response 1.8: We thank the reviewer for highlighting some important considerations relating to our comparison with Granger causality. When the term Granger causality is used, it is usually referring to the linear implementation. Transfer entropy and (this linear implementation of) Granger causality are equivalent for linear dynamics [3]. Thus, Transfer entropy is one of the many possible non-linear versions of Granger causality. This fact will not be known to all readers of the paper and we appreciate this being brought to our attention. As Granger causality was applied to this example with time-binning, our implementation of discrete-time TE on this example is a test of an equivalent non-linear

version of Granger causality. As this non-linear version of Granger causality does fail, we suspect that the reason for failure is not due to non-linearity, but rather time discretisation. We thank the reviewer for bringing to our attention the need to emphasize that there is no hidden confounding in this example.

Updated text 1.3:

1. The following has been added to the discussion:

   > It is worth highlighting in this context that TE is a non-linear version of Granger causality [3]. This makes us suspect that the reason for the earlier failures of Granger causality applied to this network was due, at least in part, to time binning and not entirely due to its inability to find nonlinear relationships.

2. The following has been added to subsection E of the results (Along with multiple other changes to this subsection):

   > As the dynamics of the network are fully captured in the three neurons of the network (we have full observability), and due to the causal Markov assumption, in the case where there is no directed connection from a source to a target, the target's present will be conditionally independent of the source's past.

Also, it would be useful to provide TE estimation results compared with Runge's (Runge et al. 2018 Escaping the Curse of Dimensionality in Estimating Multivariate Transfer Entropy) and Gao's (Gao et al. 2016 A Transfer Entropy Method to Quantify Causality in Stochastic Nonlinear Systems) methods. I understand the fundamental extension of the proposed method compared to the other methods, but it would be interesting to see how poorly the other methods perform if that is the case. Finally, how is the proposed method related with the "Instantaneous Transfer Entropy for the Study of Cardio-Respiratory Dynamics" of Valenz et al. and the "Optimization of relative parameters in transfer entropy estimation and application to corticomuscular coupling in humans" of Zhou et al?

Response 1.9: We thank the reviewer for bringing this related work to our attention.

1. The referenced paper by Runge does not present an estimator, but rather a scheme by which one can determine which processes to include within a conditioning set. It could, therefore, be used in conjunction with our proposed estimator as part of a network inference scheme. Please refer to the cover letter for more discussion on how our presented work could be utilised in conjunction with the network inference algorithms which are closely related to the methodology proposed in the referenced paper. However, given that this work does have some relevance to our paper, we have added a citation to it.

2. Gao et. al. and Zhou et. al. both present approaches to estimating for TE on continuous variables in discrete time. As such these works are not applicable to event-based data (which are normally represented with binary variables in discrete time).

3. Valenz presents a model-based approach to estimating TE, where this model is chosen to suite heartbeat dynamics. Being model-based for a different system, it is not applicable here.

Updated text 1.4: "(see Runge [77] for a methodology for reducing the dimensionality outside of network inference)" has been added to the discussion.

Minor

This is a personal preference but in general X in causal terminology is used for potential cause/source and Y for target variables. Nevertheless, authors are consistent with the way they define X and Y, so this is not a thing that I insist on changing.

Response 1.10: We appreciate that this might cause confusion for some readers. We have decided to maintain consistency with the TE literature, where Y is used for a source and X is used for a target.

Line 100: "of the of the" repeated twice

Updated text 1.5: Changed to "of the"

Line 856: "through the mediating variable $Z \rightarrow X$.

Updated text 1.6: The phrase has been changed to: "through the mediating variables $\boldsymbol{\mathscr{Z}}_{<t}$ and $\mathbf{X}_{<t}$."

Line 893: "then, we then find" — delete one "then"

Updated text 1.7: ", we then find"

Line 1118 reads weird

Response 1.11: We agree that it reads weird. Thanks for spotting this.

Updated text 1.8: We have changed the line from "The simulation approach used follows very closely that presented in" to "We devised a simulation approach which follows very closely that presented in"

2 Reviewer 2

Review for "Estimating Transfer Entropy in Continuous Time Between Neural Spike Trains for Other Event-Based Data"

In this manuscript, the authors present a method for estimating transfer entropy from discrete event time series data. They develop the method using k-nearest neighbors estimates of relevant entropic terms. The method is shown to produce better estimates of transfer entropy as compared to other typical methods that discretize the data using time bins, making use of a series of constructed test examples. (One test example is a pre-existing model of bursty neural firing.) The authors also construct a novel method for creating surrogate shuffled datasets to be used for significance tests, identifying and curing a problem that incorrectly breaks conditional correlations when using naive time shuffling approaches.

Overall, the manuscript is well written and convincingly makes its case that the proposed method is better than naive binning procedures for estimating transfer entropy in these cases. The method would be immediately applicable to common spiking datasets in neuroscience.

The development is comprehensive mathematically, though I wonder if a bit more discussion about biological applications would be useful for this audience. For instance, when does the proposed method fail? Does the amount of data required for resolving connectivity depend on the amount of noise in the system? Do you expect the method to successfully scale up to cases with, for instance, hundreds or thousands of neurons? I realize these may only be addressable in future work, but any preliminary thoughts here would be useful.

Response 2.1: We agree that it is important to test the scaling of our results with respect to dimension and available data. We thank the reviewer for highlighting this important issue. We would point out, however, that although the scaling of our ability to estimate TE as well as test for conditional independence is within the scope of this paper, testing the scaling of network inference is not. Please refer to the cover letter for our position on why this is the case.

Updated text 2.1:

- New experiments were run to test the scaling of the conditional independence test in the number of conditioning processes (which, as confounders also simulate the presence of noise) and amount of available data. The results of these experiments are described in the new **Results subsection D**.
- We have also added to the Discussion, as noted in our comment to all reviewers, that this conditional independence test can be embedded in higher level algorithms for network inference, and that remains to be tested in future work. The new paragraph of the discussion reads:

  > We foresee that one of the more useful applications of the conditional independence test that the combination of estimator and surrogate generation scheme provides will be network inference. Strictly speaking, statistical methods such as these produce effective network models which are not generally expected to provide precise matches to underlying structural connectivity. Under certain idealised circumstances though, as implemented in our experiments, the two can be expected to match, and this provided for the important validation of our methods on these small networks. The extent to

which our method can be validated in this manner on larger networks, and more importantly the extent to which the network models it infers correlate with underlying structure outside of such idealised conditions, remain open questions though. This is an intended focus of future work. Indeed, the inference of the connectivity of spiking neural networks from their activity is an active area of research [73, 74] which includes recently proposed continuous-time approaches [75, 76]. Any conditional independence test will suffer from the curse of dimensionality. This means that performing effective network inference requires pairing the conditional independence test with a suitable (conditional-independence-based) network inference algorithm which reduces the dimensionality of the tests. A variety of such algorithms exist [61]. The greedy algorithm [7, 47], which has already been validated for use in combination with TE (for different types of dynamics on larger networks), holds particular promise. It was recently shown by Das and Fiete [48] that popular existing approaches to the inference of spiking neural networks, such as generalised linear models and maximum entropy-based reverse Ising inference, had very high false-positive rates in instances where the activity of unconnected neurons was highly correlated. Given our focus on demonstrating that our conditional independence test is highly robust to strong pairwise correlations despite conditional independence, we believe that the work presented in this paper holds great promise towards making progress on this important issue

Other comments:

Eq (1) seems to be missing an average over possible states and histories? (Or if not, a brief comment would be nice to orient the reader.)

Response 2.2: Thanks for pointing out our lack of clarity here.

Updated text 2.2: "The average here is taken over time, as opposed to possible states and histories (whilst both formulations are equivalent under the assumptions of stationarity and ergodicity). " Has been added just below this equation.

The notation of Eq (3) and (4) was confusing to me before reading the Methods. Specifically, it is not immediately clear that λ depends on time. (Should t be t_i?)

Response 2.3: The use of t is chosen here to emphasize that the rates are defined for all time (not just at target spikes). The rates are presented as a function of x_i, which are the time points of the target spikes.

Transfer entropy rates are quoted as nats/second (e.g. in Fig 2), but it was not immediately clear to me where the timescale of seconds came from.

Response 2.4: The timescale comes from the timescale that we choose for the normalization variable T.

Should N_S/N_X in Fig 2 be N_U/N_X?

Response 2.5: This has been fixed. Thanks for spotting this typo.

Text in figures is extremely small and hard to read.

Response 2.6: We agree with the reviewer. The text in all figures has been enlarged.

line 368: "a p value of zero to every single example..." This isn't evident from Fig 7—it looks like there are some nonzero p values for $\sigma_D = 5e - 2$?

Response 2.7: Thanks for spotting this clear error. We have rerun all experiments for this resubmission. In the rerun, it happened by chance that there were no nonzero values.

Typo: line 100: "of the"

Updated text 2.3: We have removed the repeated occurrence of "of the".

3 Reviewer 3

In this manuscript, the authors propose a continuous time framework for estimating information flow in terms of Transfer Entropy (TE) between two point processes. Building upon a recent continuous time formulation of TE [1], they propose a concrete method for assessing information flow between the two considered processes mediated through discrete events in time. To that end, they introduce a Bayesian inversion for conditional TE rates that simplifies the required computations, and employ (already established) K Nearest neighbors (k-NN) empirical estimators for estimating the ensuing entropic terms. They further introduce a permutation method to generate surrogate time series for testing conditional independence in settings where spurious correlations may result in inaccurate predictions of information flow. For proof of concept, they demonstrate the bias, variance, and consistency of the proposed estimator on increasingly challenging trivial settings of synthetically generated time series, and contrast the performance of the estimator against the discrete time TE estimator performance. Finally, they demonstrate the performance of the framework on inferring synaptic interactions on synthetically generated data from a small biologically inspired network (the pyloric circuit of the crustacean stomatogastric ganglion), with self-sustained rhythmic bursty activity. I consider the topic of article interesting and well timed, recognising the issues arising from time discretisation of spiking activity data, especially for addressing the problem of network reconstruction. In this setting, time series of hundreds of neurons are usually available, and the binning of firing times would result either in imprecise estimations (when large time bins are employed) or in high computational demands (when small time bins are used). TE is a widely used tool employed for estimating information flow in brain activity data, and the present article improves the estimation performance of TE methods when one has to consider spiking activity data, by avoiding the complications arising with time discretisation. Considering the problem of network reconstruction from such data, to my knowledge, there is only a limited number of previously published studies that circumvent the issues of time-binning by considering inter-spike intervals instead of spike times.

I found the methodology followed in the article sound and original and I consider the whole work an essential contribution to the field. However, the authors fail to address two essential points:

how their framework scales with network dimension in terms of computational demands and required amount of data (either with numerical experiments or with theoretical analysis)

Response 3.1: We agree that it is important to test the scaling of our results with respect to dimension, computational demands and available data. We thank the reviewer for highlighting this important issue.

Updated text 3.1: New experiments were run to test the scaling of the conditional independence test in the number of conditioning processes and available data. The results of these experiments are described in the new **Results subsection D**. We have also added a note on computational demands to the end of results subsection E.

some discussion or comparison on how the proposed method compares to existing approaches for inference of network interactions from spiking activity data.

Response 3.2: We thank the reviewer for highlighting the importance of comparing our proposed methodology with pre-existing approaches. The principal focus of this work was not the inference of network interactions, but rather the development of an estimator of TE and an associated surrogate

generation scheme. Please see the cover letter for more details. The conditional independence test resulting from this estimator and surrogate generation scheme can be used for network inference in conjunction with other algorithms. However this is beyond the scope of this article. It is certainly necessary to test how this conditional independence test compares with other such tests on spike trains. The authors are unaware of any previous work deriving such a transfer-entropy-formulated conditional independence test specifically for spike trains. Moreover, any test for conditional independence between the present of a process and the history of another is just a test for non-zero TE. We have thoroughly tested the previous approach to testing for non-zero TE on spike trains in instances where conditional independence or otherwise can be expected.

Moreover, I consider that parts of the article were hastily written and the authors should revise the language to improve readability. Although I really appreciated the schematics accompanying the manuscript, which considerably help the reader to comprehend the explained concepts, I consider that the figures demonstrating the results from the numerical experiments require improvement. Finally, I consider that the main experiment requires a more systematic numerical study. Overall I suggest the manuscript for publication, given that the aforementioned issues and the detailed points for clarification/improvement have been sufficiently covered. Here I mention the points that require improvement and/or clarification in what follows on a point-by-point basis:

Response 3.3: We respond to each of the above comments where they are elaborated on in more detail below.

General remark on the plots: I found the plots difficult to read. I would suggest the authors to increase font sizes and line widths.

Response 3.4: We agree with the reviewer. The text font sizes and line widths in all plots have been increased.

Results A

In Fig. 2 and 3 the authors compare the mean and variance of the estimators for increasing number of target events NX, however, they employ different number of independent realisations among which they compute their statistics for each NX (Line 194: 1000,100,20 and 20 tested process pairs). I propose to stick to a single number of realisations among all the comparisons to be able to draw conclusive remarks on the variance of the estimator for increasing number of target events.

Response 3.5: Please refer to response 1.6.

I would propose the authors to present the results of Figure 2 and 3 in a single plot or at least on different plots with same axis scaling. Since the scaling of the y-axis in these two series of plots is different (the discrete time estimator is plotted on a smaller y-axis scale), it is difficult for the reader to easily visualise the claims regarding the variance of the estimator. For example, in Line 209 the authors claim that the variance of the discrete time estimator is comparable to the variance of its continuous counterpart, but as far as I can tell from the plots the variance of the discrete estimator is considerably lower. Also I would expect the lengths l and m of the embedding of the discrete time

estimator to match the length of the embedding of its continuous counterpart, i.e. $l \times \Delta t = \langle \text{ISI} \rangle \times l_X$. Thus for increasing Δt I would suggest presenting results with embedding lengths that match the relevant results of the continuous time estimator for a fair comparison.

Response 3.6: We agree with the reviewer that greater clarity is achieved by having figures 2 and 3 share the same scaling. This has been implemented in the revised paper. We further agree that it would be interesting to observe how the discrete-time estimator performs with an embedding length that spans the same average distance as the continuous-time estimator. This experiment was run and the result is presented in the new figure 13 of Appendix B).

In Figure 2 for increasing number of target events (for 105) the bias of the estimator slightly increases when the ratio NU /Nx is large. Do the authors 2 have an explanation for this? For lower ratios NU /NX this bias does not arise.

Response 3.7: This behaviour was the result of a minor bug in our code. This bug has been fixed and all experiments have been rerun.

Specifically, in earlier development work, in order to rule out the potential for floating-point related numerical errors, we added a very small ε to the radius searches in lines 8 and 13 of algorithm 1. Due to our oversight, we forgot to remove this later. This slightly increased radius would allow, in a tiny fraction of cases, an extra point to be added to the set of neighbours when performing these searches, which resulted in the small observed bias.

We thank the reviewer for highlighting this as it resulted in us resolving this bug.

In Fig. 2 what would happen if you would increase the number of embedding length for both time series? Do the properties of the bias and variance still hold? I would like to see either results from numerical experiments or at least some statement that increasing embedding length did not influence the outcome (if that was the case). Also in a more general setting what would be the advice on the choice of the embedding lengths?

Response 3.8: We agree with the reviewer that it is important to validate the estimator for longer history lengths. The new figure 12 in Appending B contains a plot with histories of length 3. The setting of the history length is usually an integral part of the higher-level network inference algorithm that could be associated with this estimator. We have added a sentence referring the reader to work where this is discussed.

Updated text 3.2: The following has been added to results section B.

> See [49] for a discussion on how to set these embedding lengths.

Similar question for the number k of nearest neighbors. How would one choose in practice the number k?

Response 3.9: Previous authors have studied the impact of the parameter k on this class of estimators. Thanks for highlighting the need to point the reader to this work.

Updated text 3.3: The following has been added to results section B.

> this reflects known results for variance of this class of estimators as a function of k, where generally k above 1 is recommended [50].

Results B

See first point of Results A regarding the number of realisations.

Response 3.10: Please refer to response 1.6.

Figure 4b: Lines: 247 - The authors claim that for $l_X = 3$ the estimator converges to the true value, however as far as I can see from the plots, the estimator seems to have a slight bias both for $l_X = 1$ and $l_X = 3$, and converges to the true value only for $l_X = 2$. Do the authors have a more detailed explanation for this phenomenon (similar to the explanation they provide for $l_X = 1$)?

Response 3.11: The reviewer is correct in spotting that, in the original manuscript, for $l_X = 3$ the estimator has not quite yet converged for the size of data on which we were testing it. We have since re-run the experiment out to 1 million target spikes and have replaced figure 4b with the results of this experiment. Below we have included the identical plot, but with smaller line widths so that the reviewer can observe the convergence more closely.

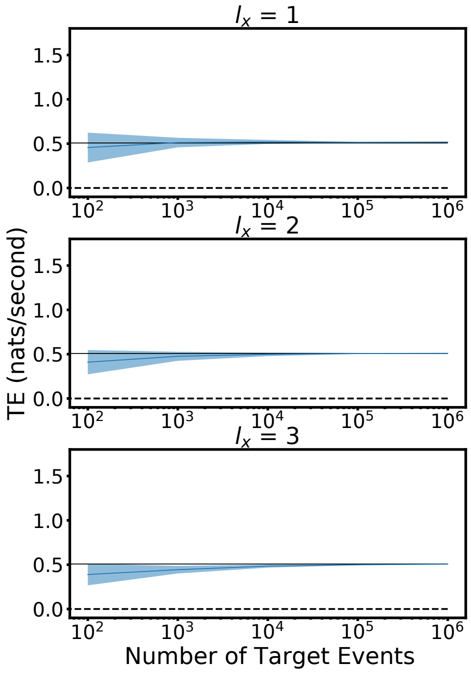

Similar comment here to the one mentioned in Results A, are the results indeed qualitatively similar when the embedding length l_Y increases? From Fig. 4b it seems that at least the embedding length of the target l_X contributes a small bias. Does increasing l_Y have a similar influence? It might be helpful to either present some numerical results (in the appendix) or write a short statement on that.

Response 3.12: As only the most recent spike in the source contributes to the dynamics of the example, increasing the length of the source embedding will not affect the underlying true value of the TE. However, it is possible that it could affect the bias and rate of convergence. We repeated the experiment in the figure with $l_Y = 3$. The results are shown in figure 14 of appendix B.

Results C

Please mention in the appendix how you calculate the p-values.

Response 3.13: We thank the reviewer for spotting this omission.

Updated text 3.4: The following text has been added to the Methods.

> The p values are calculated by constructing $N_{\text{surrogates}}$ surrogates by the algorithm just described. The TE is estimated on these surrogates and compared to the TE estimated on the original embeddings. The p value is then the number of estimates on surrogate embeddings which were larger than the estimate on the original data divided by the total number of surrogates.

Fig. 7: I would recommend to present the results on a logarithmic y-axis, so that the small p-values become visible. With the current axis it seems that some zero flow TEs resulted in zero p-values, although, I assume they were larger than the cut-off p-value. Currently, the reader cannot clearly draw a conclusion that the claims in the article are accurate. I would suggest showing here p-values of individual experiments with logarithmic y-axis, overlayed by a plot that captures the summary statistics (i.e. the box plot used here), with an additional line indicating the cut-off p-value.

Response 3.14: We agree with the reviewer that showing the p-values of the individual experiments along with a line showing the cutoff p-value add much greater clarity to these plots. We thank the reviewer for these useful suggestions. We tried implementing the suggested logarithmic y-axis. However, it is our opinion that such a change reduces the clarity of the plot. In the case of zero information flow, the ideal goal for conditional independence tests is a broad range of p values around 0.5 . This can be clearly spotted on a linearly scaled y axis but is much more difficult to observe under logarithmic scaling.

Here as a suggestion I would propose the authors to draw a small schematic with the 3 processes (D1, D2, and M) and show connections from M to D1 and D2 in Fig 7. Also probably annotate the x-axis of the existent plots with the corresponding label of each connection, (i.e $D1 \rightarrow D2|M$ and $M \rightarrow D2|D1$), for better visual clarity.

Response 3.15: We agree that such a schematic would aid the reader. As such, it has been included as part of figure 5. We have also incorporated the suggested labelling into figure 7.

Results D

Response 3.16: A note to avoid confusion: This subsection is now Results E.

For the continuous time estimator the authors use embedding length of $l_X = 1$ interval. I am wondering whether the results would improve by considering lengthier embeddings that would essentially span one whole burst event.

Response 3.17: The experiments presented in this have now been re-run with the longer embeddings of $l_X = l_Y = l_Z = 3$. The appendix also contains experiments for lengths 2 and 4. However, given that each burst can contain up to around 10 spikes, including this number of intervals in each history (giving us a total dimension of 30) would be infeasible. Please see the new Results subsection E for experiments showing the scaling of our approach with dimension.

Given that this is the main numerical experiment of the manuscript, I would expect a more systematic assessment of the performance of the framework for different hyper-parameter values, i.e. NX, lA etc. I would propose a more systematic numerical study especially in terms of data requirements.

Response 3.18: We do not believe that this is the main numerical experiment of the manuscript. Please refer to the cover letter for more details. However, we do agree that extra tests using different hyper-parameters are informative. These tests have been added to the appendices.

Updated text 3.5: Figures 17 and 18 of Appendix B show the experiment run with different values of the embedding lengths as well as N_X.

Same comment here for the presentation of results as in Results C. Moreover, I am wondering whether the predicted TE values reflected the strength of the underlying synapses.

Response 3.19: In terms of the presentation of the results, we have included most of your suggestions made for Results C. Please refer to our response there (response 3.14).

In terms of the predicted values reflecting the strength of the synapses, while we expect this to often be the case, it will not hold in all circumstances. For instance, in cases where a periodic source is driving a target, the TE value will be much lower than the strength of the synapse might suggest. This is due to a large portion of the influence of the source being predictable from the history of the target.

Response 3.20: Please refer to response 3.14.

Methods

I found this part of the manuscript clearly written.

In line 722 the authors mention that the NU sample points are collected randomly. I would like a more precise definition on the selection of the U points. Are they selected randomly from a uniform, Gaussian or some other distribution?

Response 3.21: We thank the reviewer for spotting this omission. When rerunning the experiments for this resubmission we decided to place the points at fixed intervals as this would provide a more even sampling of histories. This choice does not have a significant impact on the results.

On the method for surrogate generation, although I found the approach sound theoretically, I wonder how easy it would be in practice to construct these surrogates in a general setting for a network in the asynchronous irregular regime? In the considered circuit presented here, the neurons fire in highly repetitive manner (low coefficient of variation (CV)) and therefore constructing samples with similar $x^1_{<u_b}$ and $z^1_{<u_b}$ as the event intervals $x^1_{<x_i}$ and $z^1_{<x_i}$ is probably trivial. Yet, in a setting with more than three neurons (therefore with cardinally of set Z, $|Z| > 1$) with high coefficient of variation, one would have to identify a time point in the recording where all the conditioned neurons (Z) fire with the same timing as in $j < x_i$. My question is twofold: 1] How robust is the method when the generation of surrogates is slightly imprecise for the conditioned time series. 2] In general how robust is the method for increasing irregularities in the spike timings of the observed processes? Is there any limit of CV above which the method underperforms? Does the method require more data/lengthier recordings when the neuronal activity becomes more irregular? Could you probably quantify this relationship? Would the authors propose this method only for networks with oscillatory activity? How does the required number of sampled points NU (or the ratio NU /Nx) scale for increasing dimension in order to achieve sufficiently accurate results?

Response 3.22: We agree with the reviewer that these are very important questions.

Updated text 3.6: The new experiments in **Results subsection D** address these questions. Specifically:

- The source processes in the experiments presented there are either homogeneous or inhomogeneous Poisson processes. The homogeneous Poisson process is the maximum entropy distribution for a point process with a given average rate.
- The plots in that section demonstrate scaling both in the number of target spikes and the in the size of the conditioning set $|Z|$.

Conclusions

I miss some comparison or at least discussion with already established methods for inferring connections from spiking activity data. I think the authors should discuss what are the expected advantages of

their approach compared to existent approaches (e.g. GLMs, model based approaches)(see [2, 3] and references). Moreover I consider the authors should comment how their method compares to these two methods [4, 5] that employ a similar construct for analysing spike train data (instead of the commonly used binning). In the present article the authors need to create samples also at non-event times. Since this requirement contributes additional computational demands, especially for increasing dimensions, do the authors think that this additional sampling would be able to capture additional information that these two approaches would fail to capture?

Response 3.23: The focus of this paper is not on network inference. Please see the cover letter for more detail.

The authors present their framework in a setting with rhythmic bursty activity, and claim that the continuous time TE framework is able to capture relationships along the different relevant time-scales, while for the discrete time estimator there is trade-off in selecting short or long time bins. Although I totally agree with this claim, I am wondering whether the performance of the discrete time estimator would improve if one would consider a coarse grained representation of events in this setting, i.e. consider the onset of a burst as an event, thereby considering inter-burst intervals instead of inter-spike intervals, and therefore employ longer time bins for the time discretisation. Would the results of the discrete estimator improve and compare more favourably to the continuous time estimation with this consideration?

Response 3.24: Changing from spikes to bursting events or avalanches is a great idea, which we've explored briefly in the past [17]. However, time binning the avalanches will still have the same drawbacks as time binning spikes in comparison to using our continuous time approach to analyse inter-avalanche relationships.

Related to some of the previous points: I would like to see some comment, if not numerical experiments, on the computational demands of the method and how they are expected to scale with network size. What would be the limiting factor for applying the proposed framework in realistic settings with recordings comprising hundreds of neurons? I expect in high dimensions, the estimation of cross entropies with the k-NN estimator would be rather inaccurate, and would require long recording times. Is that true?

Response 3.25: We agree with the reviewer that these are pertinent questions.

Updated text 3.7: The new experiments in the new **Results subsection D** addresses these questions. The plots in this section demonstrate the scaling of the conditional independence test with the dimension of the conditioning set and the number of target spikes (which is a proxy for the recording time).

Further minor suggestions:

Here and there in the article there are missing full stops, especially when a sentence ends with an equation.

Response 3.26: We thank the reviewer for bringing this to our attention.

Line 53: I would skip the expression "as above", since it hints to network inference with TE being explained above, while it was only mentioned in line 51 pointing to some references.

Updated text 3.8: We have removed " as above".

Line 107: "of the" repeated occurrence.

Updated text 3.9: We have removed the repeated occurrence.

Line 123: I would use the expression "ignoring the inter-event interval time where the neuron is quiescent" or something along these lines. I find the "empty space" description rather nontechnical.

Response 3.27: We agree with the reviewer that this expression does sound nontechnical. Moreover, their suggested phrasing does a much better job of describing the point we were trying to make.

Updated text 3.10: "ignoring the 'empty' space in between" has been changed to: "ignoring the inter-event interval time where the neuron is quiescent"

Line 127: "doing so" sounds informal.

Updated text 3.11: "This paper presents an empirical approach to doing so" has been changed to "This paper presents an empirical approach to performing such estimation"

Line 133: something is wrong with this sentence.

Response 3.28: We agree that the sentence is awkward.

Updated text 3.12:

> This further allows us to utilise k-Nearest-Neighbour (kNN) estimators for entropy terms based these probability densities, bringing known advantages of consistency, data efficiency, low sensitivity to parameters and known bias corrections

has been changed to

> Furthermore, this allows us to utilise k-Nearest-Neighbour (kNN) estimators for the entropy terms based these probability densities. These estimators have known advantages of consistency, data efficiency, low sensitivity to parameters and known bias corrections.

Line 139: The authors are referring to "These surrogate samples.." although they only mentioned previously surrogate samples in the abstract.

Updated text 3.13: "To conclude that there exists non-zero TE between two processes a suitable hypothesis test is required. This is usually done by creating a surrogate population of processes which conform to the null hypothesis of zero TE. " Has been added to the start of this paragraph.

Line 155: please add the word "hypothesis" after the "null".

Updated text 3.14: The suggested change has been incorporated.

line 178: delete "a"

Response 3.29: This article is necessary for the sentence to make grammatical sense.

Line 203: I would replace "is dramatically better" with "dramatically improves".

Updated text 3.15: The suggested change has been incorporated.

Line 211: last sentence is unclear.

Updated text 3.16: This has been changed from

> It would appear that, in the case of these independent processes, it is consistent.

to

> We do observe this estimator converging to zero (the true value of the TE) as we increase the available data. This would suggest that it might be consistent on this specific example. However, we will shortly encounter an example where this is not the case.

Line 215: "a homogeneous"

Updated text 3.17: The suggested change has been incorporated.

In the titles of the plots in Fig. 2 the ratio is mentioned as NS/NX, while in the figure caption as NU /NX.

Response 3.30: This has been fixed. Thanks for spotting this typo.

Figure 4c please extend the y axis a bit below zero so that the estimation at zero at the upper subplot becomes more visible.

Response 3.31: The y axes of both figure 4b and 4c have been extended below zero.

Please check that you are consistent with use of tenses within a section/paragraph of your text.

Response 3.32: We thank the reviewer for highlighting this. We have made our best effort to remedy such mistakes.

Figures 7 and 8: I would suggest presenting the distributions of results with a strip plot overlayed with a plot that summarises their statistics (like the box plot).

Response 3.33: Please refer to response 3.14.

Line 559: space between words “bias” and following eq.. same at 561 for “variance”.

Response 3.34: These words are being used as the names of functions. The brackets that follow them express their arguments.

Line 563: I would add the phrase “in the limit of infinite data points”. Although it is explained at the next sentence it makes the statement more complete.

Updated text 3.18: The sentence has been changed from

> Another important property is consistency, which refers to whether, in the limit of infinite data, the estimator converges to the true value.

to

> Another important property is consistency, which refers to whether, in the limit of infinite data points, the estimator converges to the true value.

Line 603: Please give the full name of the abbreviation KSG.

Updated text 3.19: “KSG” has been replaced by “Kraskov-Stögbauer-Grassberger”

Line 643: I would suggest to use a different font type to denote the set X, probably

Response 3.35: We thank the reviewer for this suggestion. Much care and thought was put into finding notation for this paper that would be as clear and consistent as possible. We decided to only use special type for the set $\mathscr{X}$ as it is a set of point processes, whereas X and Y are just single point processes. We worry that using special type for X will make this distinction less clear.

[1] Richard E Spinney, Mikhail Prokopenko, and Joseph T Lizier. Transfer entropy in continuous time, with applications to jump and neural spiking processes. Physical Review E, 95(3):032319, 2017.
2 Yury V Zaytsev, Abigail Morrison, and Moritz Deger. Reconstruction of recurrent synaptic connectivity of thousands of neurons from simulated spiking activity. Journal of computational neuroscience, 39(1):77–103, 2015.
3 Josef Ladenbauer, Sam McKenzie, Daniel Fine English, Olivier Hagens, and Srdjan Ostojic. Inferring and validating mechanistic models of neural microcircuits based on spike-train data. Nature communications, 10(1):1–17, 2019.
4 R Cestnik and M Rosenblum. Reconstructing networks of pulse-coupled oscillators from spike trains. Physical review. E, 96(1-1):012209, 2017.
5 Jose Casadiego, Dimitra Maoutsa, and Marc Timme. Inferring network connectivity from event timing patterns. Physical review letters, 121(5):054101, 2018.

4 Reviewer 4

The present study appeals to the superiority of the continuous time series analysis method over the discrete binning method in analyzing neuronal spike data using Transfer Entropy. Especially, it is interesting to note that the authors demonstrated a possibility that the discrete method has an estimation bias, which indicates that the continuous time series method may be superior to the discrete binning method. However, their statements have, at least, two important problems to make sense. Besides, the authors will be necessary to choose more balanced and rational expressions at overall literatures.

- The authors seem not to have fully considered the motivations of other researchers applying Transfer Entropy to real-world neural data, or the considerations of others in choosing their parameters. For example, they have chosen "true transfer entropy" as the ground truth. Although the authors' standing point is also valuable, what many other researchers choose to ground truth is the "true structural connectivity" and would not be a "true Transfer Entropy". Authors completely ignored this kind of wide perspective and did not evaluate structural connections at all.

  Response 4.1: The focus of this paper is not on network inference, as per our response to all reviewers at the start of the cover letter. We agree with the reviewer that much of the community regards the inference of structural connectivity to be an important problem. The conditional independence test presented here could be used in the context of a conditional-independence-based network inference algorithm in order to attempt to infer such connectivity (provided certain conditions were met). And certainly the improved performance of our estimator over the existing discrete-time estimator in establishing conditional dependence/independence would be likely to render it more useful when incorporated in such network inference algorithms. However, extending this beyond these small examples into a full network inference algorithm is a topic for future work. Please see the cover letter for more detail.

- In the choice of models and parameters, there looks like to be several biases in the direction in the authors' favor. – For example, the model's parameter choices (fluctuations in firing timing, extremely strong burstiness, extremely Strong cyclicality) looks not to be natural choice.

  Updated text 4.1: The new **Results subsection D** contains an evaluation on an example with substantially different dynamics to the examples already presented. This will be specified in more detail in response to the further points below.

Therefore, additional evaluations and mitigation of wordings are necessary.

Clear separation of different motivations to calculate Transfer Entropy

First, the authors should clearly separate the motivations of researchers applying Transfer Entropy to neuronal spike data into the following two categories.

Category 1

Some scholars, including you, have utilized Transfer Entropy's ability to quantify information flow as a property of Transfer Entropy, which is specific to information theory, but they are currently in the minority in neuroecience [22, 25, 26, 27, 32].

Category 2

The majority of researchers have utilized Transfer Entropy, as one example of many variables, to infer or compare with structural connectivity from neuronal activity [24, 28, 29, 30, 31, 31, 33, 34, 35, 36, 37]. The statement will be clear if we see reports such as Battaglia et al. (2017), de Abril et al. (2018). For researchers in this standing point, how much a variable such as Transfer Entropy is "accurately" calculated is not primary important, rather than the prediction performance of structural connections is more essential.

Battaglia, D., Guyon, I., Lemaire, V., Orlandi, J., Ray, B., & Soriano, J. (Eds.). (2017). Neural connectomics challenge. Springer International Publishing.

de Abril, I. M., Yoshimoto, J., Doya, K. (2018). Connectivity inference from neural recording data: Challenges, mathematical bases and research directions. Neural Networks, 102, 120-137.

From that point of view, this report have potential to appeal the superiority about Category 1 because authors tried to calculate Transfer Entropy more accurately. However, only the weaknesses are noticeable for Category 2 because of the lack of evaluation of structural wirings.

Based on these issues, some modifications will be necessary.

- We strongly recommend the authors to show their performance to evaluate the structural wiring.
- If the authors do not evaluate in comparison with structural connectivity, they should clearly state the weakness of this study in the abstract, introduction, discussion, etc. by writing like "The study did not evaluate the performance of the structural wiring at all"
- The abstract will be necessary to clearly state like "we did not evaluate the accuracy of prediction of structural connections, but discourse on the computational accuracy of a transfer entropy defined in report***" and "The fact that we did not evaluate the prediction performance of structural wiring is a weakness of this study".

Response 4.2: (Responding to all 3 above points) The focus of this paper is not on network inference. Please see the cover letter for more detail.

With that said, we did indeed provide several examples (c.f. existing Result subsections C and E, and now more again with new Results subsection D) where the estimator in conjunction with the null surrogate generation method was used to test for conditional dependence/independence, in specific situations where dependence would have a one-to-one correspondence with structural connectivity. Such situations were used because they are important demonstrations of the validity of a conditional dependence/independence test, and a pre-cursor to inclusion in a more general inference algorithm. The superiority of our method over discrete time TE for inferring conditional dependence/independence (and by implication structural connectivity here) was clearly

demonstrated in these examples. So, although we are not aiming at a full evaluation of incorporating our estimator into a general network inference algorithm, the head-to-head comparison with discrete-time TE for Category 2 that the reviewer wishes for is indeed present.

- Among the cited references on p. 4-5, authors will be proper to limit them to the references of category (1) and should also remove references of category (2).

  Response 4.3: The revised paper is very clear in its focus on TE estimation and conditional independence testing. It makes no claims to the inference of structural connectivity in general. It would be deterimental to the reader to remove references to this use-case of transfer entropy estimators, which indeed rely on the conditional independence testing.

- An other related point is that authors will be better state more clearly how the "true" Transfer Entropy is defined.
- Structural wirings will have physiological ground truth. But, because that Transfer Entropy itself is a human-made variable originally by Thomas Schreiber (as originally a discrete way!), the expression like "true value of transfer entropy" in the paper sounds awkward. Authors should choose more rational expressions by replacing all expression as "true value of transfer entropy" to "transfer entropy obtained in Reference A" or "transfer entropy calculated by a method B".
- If the method to define the so called "true" Transfer Entropy is close to that of the present authors' estimation method (whether it is a truth quantity or not), then it is not surprising that the estimated values are close each other.

  Response 4.4: (Responding to all 3 above points) Transfer entropy has a true value in the same way that other statistical quantities (eg: mean or variance) have a true value, given a known generating process, stationarity, and accurate estimation of the PDFs. A principal focus in the development of any statistical estimator is that it converges to this true value. If a process naturally occurs in discrete time then one can make an argument that a discrete-time definition is natural. However, spike trains are a continuous-time phenomenon. Therefore, the discrete-time approach will only provide a natural framework for the TE rate in the limit of infinitesimal bin size. In this limit, the discrete-time approach is equivalent to the continuous-time one – i.e. the same true value exists for both estimation methods. This was demonstrated in our previous work [21].

Several issues of bias inherent to the evaluation method

Comments about many places in this report including following three parts:

P.13 L.253-254

Smaller bin sizes could not be used as this caused the machine on which the experiments were running to run out of memory.

p.17 L. 355-356

We used time bins of width $t = \Delta$ 0.05 and history lengths of 7 samples for the target, source and conditional histories.

p.20 421-422

which placed an effective lower bound on the width of the time bins (as bin sizes below $t = \Delta$ 0.05 resulted in impractically large search spaces)

Expressions like these examples are inappropriate; Although authors chose observing parameter region not to see Δt smaller than Δt =0.05 with various reason, if Δt becomes smaller, we can expect the results in the discrete TE will also become better than the current statement. Since the "continuous" TE algorithm uses information from the all sampling data of $\Delta t = 0.01$, it's also no surprise that continuous TE algorithm can get good results.

- Because authors seem not to have sufficient computational environment to calculate discrete TE for $\Delta t = 0.01$ using long data, the authors will be also impossible to reject the possibility the discrete TE gives the "true" value for the shortest $\Delta t = 0.01$.
- When the length of the data is shorter, there is no reason why the calculation should not be possible for $\Delta t < 0.05$. Authors will be better to do effort to show results of $\Delta t = 0.01$.
- Therefore, final conclusions from this study will be currently proper to be expressed in these two terms.
- When people use discrete TEs, they are better to use the shorter bin size (ideally, the inverse of sampling rate).
- Continuous TE is an efficient computation with reducing necessary computational memory than discrete TE. This solution potentially solves many issues existing in this current report simultaneously.

Response 4.5: Reponding to all of the above comments so far in "Several issues of bias inherent to the evaluation method":

- Time discretisation is a lossy transformation (it is impossible to reconstruct the process in full detail after discretisation). This implies that it will be impossible, in all cases, to estimate the true value of the TE after such a transformation.
- There will always be a maximum total number of bins (across all processes) that can be used. This is for two reasons. Firstly, the machine on which the analysis is running will run out of memory at a certain point. Secondly, the number of possible history permutations grows exponentially in the number of bins being used. The discrete-time estimator operates by estimating the probability of spiking given each different history permutation. This requires multiple occurrences of each of these permutations. If we use too many bins then we are likely to only see each history once or never. To put some numbers to this, if we allocate a total budget of 30 bins across all processes,

then the total number of potential history permutations is $2^{30} \approx 1$ billion. This means that our recorded process will need to be of the order of 1 billion times the length of the bin width. If we used a bin width of 1 millisecond, then we would need a recording on the order of 11 days, which is incredibly rare in neuroscience. The restricted bin budget across histories implies that there is a hard tradeoff in choosing the bin width. We emphasise that this trade-off is not only in memory requirements (which the reviewer focusses on), but also in validly estimating the PDFs in this large state-space.

- Following on then, small bin widths do indeed allow one to capture more fine detail. However, they will limit the length of time over which the history can extend. In our example of the pyloric network, the simulation was conducted with a bin width of 0.01 milliseconds (the discrete-time estimator was used with a bin width of 0.05 seconds). The inter-burst intervals in this example were around 1 second. Each burst lasted around a quarter of this time. If we used a bin width of 0.01 milliseconds, this would fully capture all the time-precision of the model (this is what the reviewer seems to suggest, although they stated 0.01 sec). However, given that we have to embed three processes, we will not be able to have these embeddings extending over longer than 0.1 milliseconds (assuming we restricted ourselves to less than 30 bins). This length of history will be woefully inadequate at capturing the dynamics of this example. The variance in the onset or length of bursting was orders of magnitude larger than 0.1 milliseconds. We could try aiding the discrete-time approach by measuring the average time length between the start of bursting in the target and the most recent end of bursting in a source. We could then set a time delay on the history, such that it fell over the average point of where the source stopped bursting in the instance that the history is constructed at the first spike of a target burst. However, because the variance in the length and timing of the bursts is so much larger than the length of the histories, a source spike will almost never fall within the observed history. Indeed, given that the firing rate of these neurons is around 5 to 20 Hz, regardless of the lag one defines, spikes will almost never fall within a history window of 0.1 milliseconds. Our selection of 0.05 sec is much more closely aligned to the firing rates here.

  Although we have used values specific to an example from the text, it should be easy to see that this is a general problem that will affect the discrete-time estimator in most cases. Moreover, it is insurmountable: no amount of parameter tuning will provide a general solution.

- Finally, to provide a concrete simulated example of this effect, we reran the experiments for figure 4c but included bin sizes as small as 0.01. Due to our budget of 25 bins, we restricted the histories to be no longer than 12 bins. Specifically, the history lengths were the minimum between 12 and $1/\Delta t$. The history dependence on the source was confined to the previous second from any point. However, in the cases of very small bins ($\Delta t < 0.1$) the history embeddings would not extend over this entire interval. In order to assist the discrete-time estimator, we implemented a time lag on the source history embeddings such that the source embedding would be centered around the bin 0.5 seconds from the target present bin. This was done as the source influence peaks at 0.5 seconds behind the present (see figure 4a). The results of these experiments are shown below. We notice that, for very fine bin widths, the value of the TE becomes very small. This is because the histories cannot cover the entire interval over which the source history is relevant. We also ran experiments without the lag described above, the results of which are worse than those shown here.

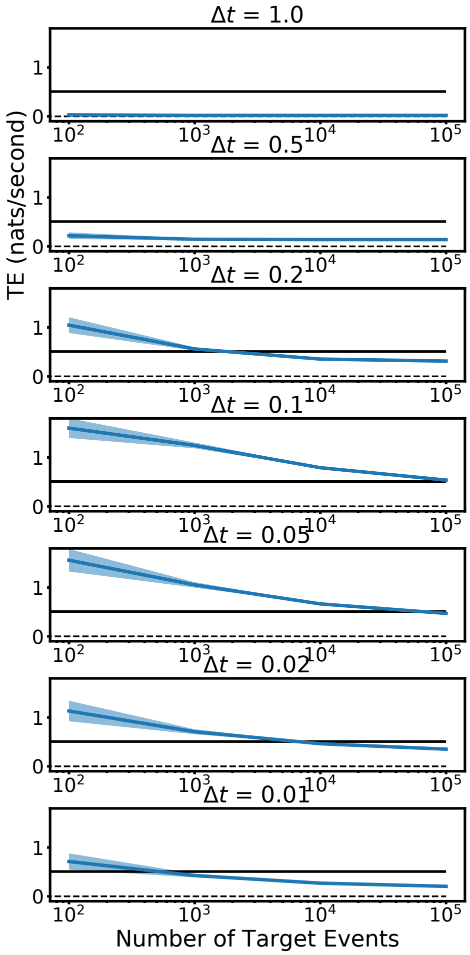

P.13, L. 258

At a bin size of t = 0.2 it converges to a value less than half the true TE. Moreover, its convergence is incredibly slow. At the bin size of t = 0.1 it would appear to not have converged even after 1million target events, and indeed it is not even converging to the true value of the TE. The significance of the performance improvement by our estimator is explored further in Discussion.

The expression of not converge will be quantitatively too much strong statement (especially in the current state of limited observations). The expression of converges slowly would be more appropriate. The emphatic expression Very will be also not recommended.

Response 4.6: We disagree with the reviewer. The plots in Fig 4c clearly show that the estimate for 0.1 bin width have not converged. They are converging slowly, but in comparison to the other parameter settings and the continuous-time estimator, they have clearly not converged (dropping from 0.55 nats/s to 0.4 nats/s from $N_X = 10^5$ to 10^6 spikes).

P.2, L. 14

This approach has numerous drawbacks: it is very biased, it cannot capture relationships occurring on both fine and large time scales simultaneously, converges very slowly as more data is obtained, and indeed does not even converge to the correct value.

Because there are many limitations and insufficiencies in the computational model and parameter selections in the current evaluations, expressions like this part is improper. The paper needs to be revised well in this regard.

Response 4.7: Please refer to response 4.5.

Abstract and Introduction

the current approach to TE estimation on such event-based data via discretising the time series into time bins: it is not consistent, has high bias, converges slowly and cannot simultaneously capture relationships that occur with very fine time precision as well as those that occur over long time intervals.

A discrete-time TE estimator cannot capture both of these classes of effects simultaneously.

The statement sounds that continuous TE can simultaneously capture both long time scale interactions and short time scale interactions. However, if we compare the raw data to jittered data on a certain time scale, even for a continuous TE, then we're able to discuss only about a specific time scale even for continuous TE. Conversely, even for the discrete TE, it is possible to extract multiple time scales if we analyzed them like Wavelet analysis in comparison with Fourier transform. Let's also think the situation when keeping the bin size small and computing TEs including delays, and then people vary the degree of smoothing. Then people will be able to systematically change the focusing time scale by changing the scale and size of the time window in which you search for peaks. Solutions will exist in myriad ways.

Response 4.8:

- We are unsure of what the practical benefit would be of applying a large amount of jitter to one's data before analysing it. We guess the reviewer suggests that information below the resolution of the jitter is destroyed. That misses the point though that, as per response 4.5 the discrete-time estimator can only capture relationships within the predefined $k \times \Delta t$ range and predefined source-target delay; the continuous-time estimator can capture relationships at multiple scales within a larger range due to its focus on inter-spike intervals.
- If the data is sampled with low sampling rate (roughly equal to the bin width that would be used in the TE analysis) and the timescale of history effects is small (on the order of, say, five bin widths) then the discrete-time approach will perform well in this specific scenario (although there is no reason to believe that it would perform better than the continuous-time approach). However, such a scenario is far from typical. Many micro electrode arrays that are used to measure neural activity sample at 30kHz. History correlations in spike trains can extend (as in our example of the pyloric circuit) over intervals on the order of 1 second. By applying the

arguments which we presented in response 4.5, it is easy to see that it would be impossible to capture the full resolution offered by the recording device and all history effects using the discrete-time approach.

- The discrete-time approach can easily be adapted to estimate a TE value based on different time scales. This can be done by adjusting the bin width or by applying some of the suggested approaches. There are, however, two issues here.
    1. A lossy transformation will still have been applied to the data, implying that the resulting estimator is not consistent.
    2. The reviewer's mention of how "people will be able to systematically change the focusing time scale" highlights *precisely* the issue which we are trying to fix. We are proposing a method which can capture different time scales *simultaneously*. It produces a single estimate of the TE which captures the effects occurring on these different scales. The suggested approaches produce different estimates of the TE which capture the relationships occurring on these different time scales separately.

p.52., Table4

There are several important problems with the mathematical models used in the evaluations on which all the arguments are based, and a radical solution is needed in this regard.

The problems are mainly the following three points:

1. There is no explicit fluctuation in spike timing even authors gave fluctuations for intensity.
2. bursts are too strong.
3. the oscillation of membrane potential is too strong.

Unless we look at the evaluation under conditions where these three factors selectively do not exist, we cannot speak to the validity of the results.

About point1:

The situation that suitable fluctuations are not contained in the ISI is fairly unrealistic or extremely specific as a model of an actual nervous system.

In the case fluctuation does not exist for ISIs, the accuracy will inevitably increase with smaller bin sizes, an unrealistic system as the subject of the analysis. In the discrete analysis, the the appropriate bin size will be normally selected after observing the variability of the actual ISIs. This would not be a realistic analysis unless the analysis assumes that spike timings variate at time sizes larger than the bin size.

So the authors need to add randomness into the ISI (Time constant) and analyzed the data again. The ISI histogram will be good be shown after the additional evaluations have been reassessed.

Response 4.9: The network in this example is chosen to follow very closely the example presented by Eve Marder and colleagues, where the underlying conditional (in)dependence properties are known to be very difficult to infer. We made very few modifications to the parameters of this model (reasons as follows), and retain the dynamical properties that make it a difficult example. We note that Marder et al. reported the dynamics of the model to closely follow real recordings; the main point though is that this example was chosen because it was established as a difficult example for these measures.

The reviewer is concerned about the apparent absence of fluctuations in the ISIs in the model. The reviewer may be referring directly to the original Eve Marder paper, which did not have noise in the ISIs. One of the modifications we had already made to the Eve Marder model was to add some noise to the current term, which then resulted in there being noise in the ISIs – as the reviewer highlights this is important. Visual inspection of Figure 9 clearly shows noise in the ISIs. Moreover, we have included a histogram of the ISIs for the PY neuron on one of the runs. It is clear from this histogram that there are fluctuations in the ISIs.

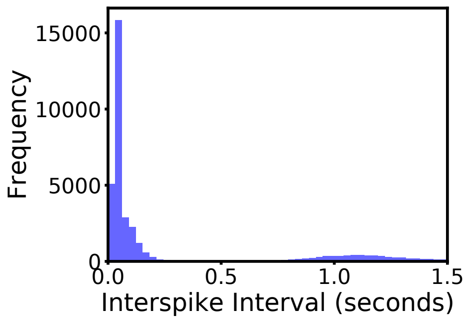

To see the performance on models with significantly higher variability in ISIs again, please see the earlier examples in the paper, specifically the new **Results subsection D**.

Updated text 4.2: To further validate our approach, we have added a new experimental setup which is presented in the new **Results subsection D**. This setup was constructed specifically to exhibit a very different regime of dynamics to that presented in our study of the pyloric network. The examples presented in this new subsection do not contain bursts and are aperiodic. Moreover, some of the examples contain sources which are generated as homogeneous Poisson processes. This implies that they have very large variation in their interspike intervals.

P.22, L. 468

In real-world applications, where the ground truth is unknown, there is no principled method for choosing which resulting TE value from the various bin sizes to use.

This is another statement that is relevant to the point made above.

Response 4.10: Please refer to response 4.4.

About point2

The simulated neurons are too much bursty. Evaluations with regular cases will be also necessary.

It should be stated that there are some systems in which bursty cells impact to the representative dynamics, but in the mammalian brain (and perhaps in your brain) they are more minor in the overall cell population than the case of the authors' model.

Example:

Bartho, P., Hirase, H., Monconduit, L., Zugaro, M., Harris, K. D., & Buzsaki, G. (2004). Characterization of neocortical principal cells and interneurons by network interactions and extracellular features. Journal of neurophysiology, 92(1), 600-608.

Therefore, simulations in situations where regular cells are in the majority should also be performed. The text should also be written in recognition of the fact that the evaluation is done in a specialized situation where bursts are strong.

Response 4.11: Please refer to response 4.9 and updated text 4.2 regarding the new additional simulations that have been included.

About point3

Another clear feature of the model's activity is the presence of a strong oscillatory component.

The current behavior of spikes are mostly dominated by periodic oscillation not by neuronal interactions. The property of the current example will also generate a strong bias. If the activity is strongly cyclical, where in each discretized bin (near the front, near the back, etc.). Therefore, a bias is likely to arise as to whether a spike will enter the system. The strong oscillation itself would be a confounding factor. For this reason, it is recommended that the authors also need to evaluate the simulation without the large periodic oscillations.

Response 4.12: Please refer to response 4.9 and updated text 4.2 again regarding additional simulations.

In any case, this situation referred to by the reviewer draws out an interesting difference between the discrete time and continuous time estimators. The confounding factor here – the periodic oscillation – is embedded in the top node of this system. In theory, conditioning on a long enough history of that node's activity would allow the estimator to interpret and include that confounder in the estimation. The discrete time estimator however is only able to condition on such activity up to a fixed specified interval into the history, and so cannot fully condition on the confounding activity and thus not differentiate well the phase of cyclical activity. The continuous-time estimator on the other hand is able to handle longer periods into the history of this confounder, differentiating between short and long times since the previous spike in the conditional variable, and therefore properly handling the confounding factor. Again, this underlines the capabilities of the continuous-time estimator with respect to the standard estimator in this case.

Other requests for new simulations

We are also concerned about the high computational cost of continuous TE.

How many neurons with how much long time recording is the method maximally applicable?

Updated text 4.3: The new experiments presented in the new results subsection D study the scaling of the proposed approach in the size of the conditioning set and the amount of available data.

Basic questions or comments

p.4 p.53 last line

I couldn't find an explanation of what t=1 means as a unit until the last sentence in the current paper.

The unit is probably ms. Did you show the information at an earlier place?

Response 4.13: In all the examples apart from those in what are now results subsections D and E no time units were used. This is due to these earlier examples being highly synthetic. In what is now results subsection E (previously subsection D) there were a two instances where we forgot to include time units. We thank the reviewer for spotting this error.

Updated text 4.4: The two instances of missing time units have been remedied.

P.52, table4

What do authors mean when the time constant is on the order of 1000-100,000 seconds?

Response 4.14: Please see table S1 in O'Leary et. al. (reference 62 in the original submission and [15] in this document), a paper which we closely followed in constructing our model. You will see parameter values of a similar range there.

P.52, l1135

c is a constant with units of seconds that was adjusted by hand

Is it ok that you easily chose it by hand without utilizing any objective optimizations?

Response 4.15: The parameter c merely controlled the speed at which the dynamics approached the steady state. The intervals over which our experiments were conducted always started after the steady state was reached. This parameter has a negligible affect on our results, and the dynamics can be seen to qualitatively match those of Marder et al., which also matched real recordings. (Moreover, in the work of Eve Marder and colleagues, which we followed incredibly closely, many parameters were tuned by hand – see references within the paper).

References

[1] Joshua D Angrist and Guido M Kuersteiner. Causal effects of monetary shocks: Semiparametric conditional independence tests with a multinomial propensity score. *Review of Economics and Statistics*, 93(3):725–747, 2011.

[2] Nihat Ay and Daniel Polani. Information flows in causal networks. *Advances in complex systems*, 11(01):17–41, 2008.

[3] Lionel Barnett, Adam B Barrett, and Anil K Seth. Granger causality and transfer entropy are equivalent for gaussian variables. *Physical review letters*, 103(23):238701, 2009.

[4] Alexis Bellot and Mihaela van der Schaar. Conditional independence testing using generative adversarial networks. In *Advances in Neural Information Processing Systems*, pages 2202–2211, 2019.

[5] Terry Bossomaier, Lionel Barnett, Michael Harré, and Joseph T Lizier. An introduction to transfer entropy. *Cham: Springer International Publishing*, pages 65–95, 2016.

[6] Abhranil Das and Ila R Fiete. Systematic errors in connectivity inferred from activity in strongly recurrent networks. *Nature Neuroscience*, pages 1–11, 2020.

[7] Clark Glymour, Kun Zhang, and Peter Spirtes. Review of causal discovery methods based on graphical models. *Frontiers in genetics*, 10:524, 2019.

[8] Dominik Janzing, David Balduzzi, Moritz Grosse-Wentrup, Bernhard Schölkopf, et al. Quantifying causal influences. *The Annals of Statistics*, 41(5):2324–2358, 2013.

[9] Jaehoon Lee, Kwanghee Jung, and Jungkyu Park. Detecting conditional dependence using flexible bayesian latent class analysis. *Frontiers in Psychology*, 11:1987, 2020.

[10] Joseph T Lizier and Mikhail Prokopenko. Differentiating information transfer and causal effect. *The European Physical Journal B*, 73(4):605–615, 2010.

[11] Atalanti Mastakouri, Bernhard Schölkopf, and Dominik Janzing. Selecting causal brain features with a single conditional independence test per feature. In *Advances in Neural Information Processing Systems*, pages 12553–12564, 2019.

[12] Christopher Meek. Strong completeness and faithfulness in bayesian networks. In *Proceedings of the Eleventh Conference on Uncertainty in Artificial Intelligence*, UAI'95, page 411–418, San Francisco, CA, USA, 1995. Morgan Kaufmann Publishers Inc.

[13] Leonardo Novelli and Joseph T Lizier. Inferring network properties from time series via transfer entropy and mutual information: validation of bivariate versus multivariate approaches. *arXiv preprint arXiv:2007.07500*, 2020.

[14] Leonardo Novelli, Patricia Wollstadt, Pedro Mediano, Michael Wibral, and Joseph T Lizier. Large-scale directed network inference with multivariate transfer entropy and hierarchical statistical testing. *Network Neuroscience*, 3(3):827–847, 2019.

[15] Timothy O'Leary, Alex H Williams, Alessio Franci, and Eve Marder. Cell types, network homeostasis, and pathological compensation from a biologically plausible ion channel expression model. *Neuron*, 82(4):809–821, 2014.

[16] Jonas Peters, Dominik Janzing, and Bernhard Schölkopf. *Elements of causal inference: foundations and learning algorithms.* MIT press, 2017.

[17] Mikail Rubinov, Joseph Lizier, Mikhail Prokopenko, and Michael Breakspear. Maximized directed information transfer in critical neuronal networks. *BMC Neuroscience*, 12(1):1–2, 2011.

[18] Jakob Runge. Causal network reconstruction from time series: From theoretical assumptions to practical estimation. *Chaos: An Interdisciplinary Journal of Nonlinear Science*, 28(7):075310, 2018.

[19] Jakob Runge. Conditional independence testing based on a nearest-neighbor estimator of conditional mutual information. In *International Conference on Artificial Intelligence and Statistics*, pages 938–947. PMLR, 2018.

[20] Rajat Sen, Ananda Theertha Suresh, Karthikeyan Shanmugam, Alexandros G Dimakis, and Sanjay Shakkottai. Model-powered conditional independence test. In *Advances in neural information processing systems*, pages 2951–2961, 2017.

[21] Richard E Spinney, Mikhail Prokopenko, and Joseph T Lizier. Transfer entropy in continuous time, with applications to jump and neural spiking processes. *Physical Review E*, 95(3):032319, 2017.

[22] Peter Spirtes, Clark N Glymour, Richard Scheines, and David Heckerman. *Causation, prediction, and search*. MIT press, 2000.

[23] Liangjun Su and Halbert White. A nonparametric hellinger metric test for conditional independence. *Econometric Theory*, pages 829–864, 2008.

[24] Xia Wang, Yongmiao Hong, et al. Characteristic function based testing for conditional independence: a nonparametric regression approach. *Econometric Theory*, 34(4):815–849, 2018.
