# Supplementary material for: Estimating Transfer Entropy in Continuous Time Between Neural Spike Trains or Other Event-Based Data
Source: PLoS Comput Biol. 2021 Apr 19;17(4):e1008054. doi: 10.1371/journal.pcbi.1008054 (PMC8084348; doi:10.1371/journal.pcbi.1008054)
Supplement: Attachment — Submitted filename: Review_TE_spikes__2nd_round.pdf [file pcbi.1008054.s012.pdf]

# Second Review Round - Comments to the authors: Estimating Transfer Entropy in Continuous Time Between Neural Spike Trains or Other Event-Based Data

November 2020

In the updated version of the manuscript and in their replies to the referees, the authors have sufficiently addressed the issues raised during the first review round by all the referees.

In particular, they have changed/clarified the overall scope and purpose of the article. They have changed the wording in their abstract and main text to indicate that the purpose of the proposed framework is *not* the inference of network couplings, but the detection of conditional independence between the considered processes. They point out that the task of network inference is left out for future work and would combine the present test for conditional independence with a suitable conditional-independence-based network inference algorithm.

The authors have moreover addressed the comments regarding hyperparameter selection of their method by additional numerical examples in the supplement, and have included a new section in the main text that demonstrates the performance of their approach for increasing data samples and conditioning processes.

Therefore, I suggest the manuscript for publication.

Here are some very minor suggestions that not do not necessarily need to be implemented in the final manuscript.

Minor suggestions:

- Line 58: I think "as *mentioned* above" sounds better here
- Line 199-201: may require rewriting for better readability
- Line 499-500: I would propose to mention Fig.8b (right) for pointing out directly to the part of the figure you are commenting on.
