# Supplementary material for: Estimating Transfer Entropy in Continuous Time Between Neural Spike Trains or Other Event-Based Data
Source: PLoS Comput Biol. 2021 Apr 19;17(4):e1008054. doi: 10.1371/journal.pcbi.1008054 (PMC8084348; doi:10.1371/journal.pcbi.1008054)
Supplement: Attachment — Submitted filename: response_to_reviewers.pdf [file pcbi.1008054.s013.pdf]

23 Decemember 2020

Article: *Estimating Transfer Entropy in Continuous Time Between Neural Spike Trains or Other Event-Based Data*

Authors: David P. Shorten, Richard E. Spinney, Joseph T. Lizier

Dear Editors and Reviewers,

We thank you for the constructive feedback and comments. In particular, we appreciate the recommendations of reviewers 2 and 3 to accept the article, and the further comments of reviewers 1 and 4 regarding improving the clarity of our message.

We now submit a revised version of the manuscript in light of these comments, and a list of responses to them. We feel that these revisions have made a positive contribution to the manuscript.

When going through the reviewers' comments point-by-point, we have copied the reviewers' previous reply text and interspersed our replies. In order to aid readability we have used the following formatting in these sections:

- When the reviewer is quoting their review in round one, we have formatted the paragraph as:
  [Reviewer in round 1] paragraph text
- When the reviewer is quoting our reply to their review in round one, we have formatted the paragraph as:
  [Response to round 1] paragraph text
- When the reviewer is writing text new to the second round of reviews, we have formatted the paragraph as:
  [Reviewer in round 2] paragraph text
- Our responses have no special formatting.

David Shorten,
for and on behalf of the authors

1 Reviewer 1

We would like to thank the reviewer for their thorough comments. These comments have revealed areas in which the paper's clarity could be improved. Addressing them has helped us produce a paper which is much more explicit on where its focus is. This will hopefully aid readers.

There are some general issues on which it appears that we were not optimally able to communicate our intent and meaning. Before attending to the reviewer's comments point-by-point, we would like to make some general comments about these issues.

General Comments

General Comment 1.1: Use of the term *conditional independence*

The reviewer has interpreted our use of conditional dependence/independence as making a *causal* claim, which was not the intended meaning. For instance, they state that "The proposed method as a dependency estimator does not work properly if acyclicity is not assumed.". When we refer to conditional dependence/independence we are not intending to make any causal claims. Rather we are simply making a statement about probability distributions. Specifically, when we say that X is conditionally independent of Y given Z, then what we mean is that the joint probability distribution factorises as:

$$p(x, y \mid z) = p(x \mid z)p(y \mid z) \tag{1}$$

This is often equivalently stated as $p(x \mid y, z) = p(x \mid z)$. This is the standard usage of the term "conditional independence". That the statistical rather than causal interpretation of the term is standard can be seen by referring to prominent scholarly texts – specifically those in the causality literature – please refer to page 10 of Pearl et. al. [15], page 214 of Peters et. al. [16] or page 10 of Spirtes et. al. [23] (or even simply the entry for "conditional independence" on Wikipedia).

It is important to note here that the conditional MI is a measure of divergence from the conditional independence condition in Eq. (1), and so—*by definition*—testing for conditional MI being > 0 (and the TE as a conditional MI) is a test of conditional dependence (as per the classic information theory text, Cover and Thomas [3]).

We acknowledge that some readers, like the reviewer, may have a causal interpretation in mind, so have added the following clarification at the first use of the term "conditional independence" in the Introduction:

Updated text 1.1: "Of course, we refer to conditional independence in the statistical $\left(p\left(x_t \mid \mathbf{x}_{<t}, \boldsymbol{\not{z}}_{<t}, \mathbf{y}_{<t}\right) = p\left(x_t \mid \mathbf{x}_{<t}, \boldsymbol{\not{z}}_{<t}\right)\right)$ rather than causal sense."

We believe that this clarification in meaning should address almost all of the comments of this reviewer.

General Comment 1.2: Use of the term *directed dependence*

The reviewer is of the opinion that the use of the term "directed dependence" implies a causal relationship, stating: "*directed* relationships *are* causal relationships". First, as per Comment 1.1 regarding dependence/independence, when we use the term we are not trying to imply causality, but instead are referring to a statistical relationship.

Second, we refer to a statistical relationship that is not symmetric (but directed). Specifically, we refer to a measure of dependence μ as directed if $\mu(X, Y) \neq \mu(Y, X)$. There is much precedent for this use of the word "directed". Kullback referred to the Kullback-Leibler divergence (which is asymmetric) as a "directed divergence" (page 7 of [10]), and the use of this term fairly common (see [11, 26, 20, 17] for examples). There are many other asymmetric statistical measures (of dependence or otherwise) which are referred to as 'directed' [14], particularly for time-series analysis [2, 21, 25]. Of particular relevance in the context of the submitted paper is the "directed information", an information-theoretic directed dependence measure for time series [13]. And most importantly for this paper, the Transfer Entropy has been referred to as a directed measure from its inception: in the original paper proposing the measure, Schreiber makes it clear that the measure is "directional" [19].

With that said, some readers may have a similar causal interpretation of "directed depenence" in mind to the reviewer, and we have made some clarifications to avoid ambiguity here. As per Comment 1.1, our clarifications on dependence/independence should largely address this. With specific reference to use of the term "directed" in this context, most of the use in the text is already clearly indicated to be statistical – e.g. the first use in the Author Summary refers to "directed *statistical* dependence". We have altered the uses of "directed" which were not clearly statistical now: "significant directed relationships" → "significant directed statistical relationships" (Results section C, discussing Fig 6), and "time-directed dependence in spike trains" → "time-directed statistical dependence in spike trains" (end of Discussion).

Point-by-Point Response

[Reviewer in round 2] I appreciate the fact that the authors added new experiments and their effort to distinguish their method from causal inference methods. However, I believe that there are still some remaining points that this is not completely clear to the reader. I believe that the way the authors presented their method and the way they used causal graph discovery on their simulated neuronal example to validate their method, caused confusion to almost all the reviewers - including myself-, shadowing the importance of the method itself. The contribution of the paper is - and as such should be presented as - a TE estimator that avoids time descritazion, combined with a surrogate scheme. However, even after the revision, to me it seems that there are still misunderstandings for the reader, with claims of the authors about detection of directed dependencies. The method, as it is, is very important and novel. This is what the authors should state. Any further attempts to connect it with directionality of dependencies, only leads to misinterpretations.

Response 1.1: We agree that the contribution of the paper centres on the presentation of the estimator, and this is the focus e.g. of the abstract and introduction. As addressed in Comment 1.1 and Comment 1.2, the claims about directed dependencies are statistical not causal and as such are correct.

[Reviewer in round 2] My remaining problem after the revision is that authors mix the assumptions needed for TE estimation, the assumptions needed for a conditional independence test and those needed for causal discovery, in their statements. Overall I do not agree with the fact that the authors advertise this method as a conditional independence test, without specification of the acyclicity assumption that is required.

Response 1.2: While the reviewer would be correct if we were presenting a test of causal coupling, given that we are presenting a test for conditional independence they are incorrect. Transfer entropy, as a conditional mutual information, corresponds to a test of conditional independence (statistical), given the available data. (Again, see Comment 1.1). This is stated, e.g. in Ay and Polani [1], two major proponents of the causal primacy view.

Acyclicity is not required for the method to provide a test of (statistical) conditional independence; it may be required for that conditional independence to correlate with causality – that's a separate issue, considered as per Methods part D discussed below. Indeed, we had already referred to acyclicity being required for the match to causality in Methods D (c.f. requirement for relationships to be represented by a DAG).

[Reviewer in round 2] After the revision, the authors added section D where they discuss assumptions for causal discovery on their neuron experiment, using their method. Nevertheless these assumptions are to make causal inference. However, authors promote their method as a conditional independence test without naming the assumptions under which this test holds. Authors mix in their statements the assumptions needed for this method to be an independence test and the assumptions needed for causal inference.

Response 1.3: Section D clarifies the assumptions required specifically for conditional dependence to correspond to causal structure. This has been made very clear in the paper, e.g. the reference to Methods D from Results: "*the conditional dependence and independence relationships in this circuit (which, as per the previous examples, are expected to match connectivity under the conditions of this experiment, see section IV D)*". As per the reference to Methods D in our Introduction, the causal structure is simply used to generate known conditional dependence/independence relationships as ground truths for validation tests for our estimator.

Let us be clear that the assumptions in Methods D are not assumptions required for conditional independence tests more generally than this. The reviewer is correct that in general these assumptions are usually associated with making causal inference, and perhaps this is why they have the impression that we mix assumptions about conditional dependence and causality. We suspect that the confusion here relates to that around dependence/independence as per Comment 1.1.

[Reviewer in round 2] Therefore, for me the following points should become extremely clear in order to recommend the paper for acceptance:

1. [Reviewer in round 2] (self-contradictory points in authors paper/response) In author's response 1.1: Authors write "Along these lines, we do not make the claim that non-zero TE implies a causal connection in general.", which is not in agreement with what they write in their paper line 216 "The comparison of the estimates to a population of surrogates produces p-values for the statistical significance of the TE values being distinct from zero and implying a directed

statistical dependence."

Response 1.4: Again, please see Comment 1.1 – the latter quote refers to *statistical* dependence only, so there is no need to temper that claim and it remains completely consistent with the former quote about causality. (You can have directed statistical dependence without a direct causal connection).

[Reviewer in round 2] In their paper, the authors do claim detection of directed dependencies when TE is not 0 (line 216). This sentence is both in contradiction to their response, and does not stand alone, for the reasons I explain in point 1.2 below. My point in my original comment was that the opposite does not hold: TE=0 does not mean lack of dependency in general, and this is what I asked authors to add in their revision, but they did not. As I explain below, acyclicity between the time series is required for the opposite to hold.

Response 1.5: As above, and again referring to Comment 1.1, the claim at line 216 (and similar elsewhere) is specifically about *statistical* dependency, so there is no need to temper that claim. As above, we test these detections of statistical dependency against what we would expect from the known causal structure (in situations where the two should be the same): "*The results are compared to the known connectivity of the studied systems; whilst we do not expect directed statistical dependence to have a one-to-one correspondence with structural connectivity in general, these experiments are designed under ideal conditions such that they would*" (referring to Methods D). The reviewer appears to be again misinterpreting our statements on statistical dependence to more generally mean causal dependence, which we have very carefully avoided implying.

Regarding the comment "TE=0 does not mean lack of dependency in general, and this is what I asked authors to add in their revision, but they did not", the reviewer has missed where we added this into our revision in Methods D: "*it is easy to construct examples whereby the present state of each of a pair of processes is determined by the history of the other process, but where the present state of each process is conditionally independent of the history of the other*" (citing [1, 7] where the reviewer's example was sourced). This is our fault for not specifically pointing out that this was added. To make it more clear again, we added the clarification "*(e.g. one can have zero TE when a real causal connection exists)*". Crucially, we pointed out in the next sentence that this violates the assumption of faithfulness to have the conditional statistical dependence structure match the causal structure.

Regarding acyclicity, as above this was already dealt with in Methods D in requiring the representation of the causal relationships as a DAG.

2. [Reviewer in round 2] (directed dependencies) On authors answer to Response 1.4: Authors write "This sentence does not imply causality. Causal relationships are not the only type of relationship. This sentence is simply reflecting the fact that TE implies that the present of the target is statistically dependent on the history of the source"
Although causal relationships may not be the only type of relationships, the *directed* relationships *are* causal relationships. I do not agree with what the authors write and with the way they update their text. Although their second sentence in the above quoted answer is correct, the updated text does not reflect their statement, as it still writes about directionality of dependencies. "TE values being distinct from zero and implying a directed statistical dependence". The moment the authors talk about directionality, it is not possible to *not* talk about causal relationship. Therefore, either they should completely drop their claims about *directed* relationships and actually write the correct sentence they gave as an answer (aka "TE implies that the present of the target is statistically dependent on the history of the source"), or they should explicitly state that causal sufficiency is assumed in the case that they want to talk about directed relationships. Otherwise the counterexample I gave the authors in my original review

will render TE inadequate for any such statements. Same for the revised sentence in point 2 updated text 1.1.

Response 1.6: Again, as per Comment 1.1 and Comment 1.2, we have made clear our use of the terms conditional indepdenence and directed, both as being distinct from causal interpretations. The reviewer says that "the *directed* relationships *are* causal relationships" – we certainly use directed to mean asymmetric (and in a Granger "causal" sense), but not in an interventional causality sense. This is all explained in our overall comments above.

3. [Reviewer in round 2] (ground truth on simulated experiment) The authors use one of their own methods to calculate the ground truth TE on the experiments with the Poisson processes. As reviewer 4 mentioned, someone, could argue about the choice of this ground truth. I would like to see a longer discussion about the selection of this ground truth. Even if we leave this aside, up to this point I could have no objection.

   Response 1.7: We refer the reviewer to the more detailed Response 4.2 on this point.

   [Reviewer in round 2] However, moving to the simulation neural example, the authors change their ground truth estimation and use the underlying known structure to evaluate their method. Now, this is causing a lot of confusion, because on one hand authors claim this is not a causal inference method, and on the other hand use it in such a way to validate their estimator. This changes the metric that the authors use to validate their method.

   Response 1.8: As the reviewer points out, at this section we move from evaluating the ability of the approach to estimate the correct TE to evalute whether it can distinguish conditional dependence (statistically non-zero TE) from independence (statistically zero TE). As pointed out by Reviewer 4 in the previous round, this is an important use-case for TE, and so both convergence to the true value and detection of conditional dependence need to be validated.

   Next, as the reviewer points out, on one hand we do not claim this to be a causal inference method and yet use the causal structure to validate our estimator. This is a subtle point; the rationale is simply that the causal structure is used to generate known conditional dependence/independence relationships for us to validate the estimator's performance. (This is why we require assumptions of ideal conditions whereby the conditional dependence structure aligns with the causal structure). We have explained this rationale in several places in the text already, e.g.:

   - in the Introduction: whilst inference "*using TE estimators such as ours are not expected to align with structural networks in general, they would be expected to do so under certain idealised assumptions ... implemented in these experiments*", providing a "*crucial validation*" that the statistical relationships "*they infer at scale are readily interpretable*".
   - in the Results: "*The results are compared to the known connectivity of the studied systems; whilst we do not expect directed statistical dependence to have a one-to-one correspondence with structural connectivity in general, these experiments are designed under ideal conditions such that they would. This provides important test cases for detection of conditional dependence and independence.*"

   [Reviewer in round 2] Please add the word "simulated" in the title of section E. As it is presented now, it can lead to overestimation of the ability of the proposed TE method to work as a causal discovery tool in real, heavily latent-confounded noisy datasets, as the neuronal activity.

   Response 1.9: We have added "simulated" in the title of this section as requested.

   Furthermore, we have added additional caveats to the 2nd last paragraph of the Discussion regarding future work investigating how the inferred conditional dependence structure may relate to underlying structure in larger, more latent-confounded networks.

4. [Reviewer in round 2] (conditional independence testing) Re the comment of the authors that all conditional independence tests suffer from dimensionality or small sample size (line 753). It is different to talk about statistical strength of a test (in that case indeed dimensionality and small sample size play an important role about the rejection of conditional independencies), and to talk about a TE estimation used to quantify a dependency. Here TE is used to quantify the dependency and the surrogate scheme to detect the p-value. The proposed method as a dependency estimator does not work properly if acyclicity is not assumed. Therefore the method can be considered only a *sufficient* but not a necessary conditional independence oracle, if the necessary assumptions are not mentioned.

   Response 1.10: The latter statement of the reviewer is of course correct, assuming the reviewer meant "causality oracle" instead of "conditional independence oracle" (again see Comment 1.1 on why the method is a conditional independence test, always, distinct from causality). We thought the requirement of conditions such as acyclicity was clear at this point in the text from the statement in that paragraph: "*Under certain idealised circumstances though, as implemented in our experiments, the two can be expected to match*"; to make this more clear again, we have referenced those conditions to Methods D.

   [Reviewer in round 2] If the proposed TE method gives non-zero value, then they conclude dependency. However, the other direction does not hold. If the TE is zero then this does not necessarily mean lack of dependency. The counterexample I explained in my review is the reason why. A correlation permutation test, given a sufficient sample size, would find a dependency between X and Y if $X_{t-1} \to Y_t$ and $Y_{t-1} \to X_t$ and $X_{ts} \to X_t$ and $Y_{ts} \to Y_t$, for all $s > 0, t > 0$. However the proposed conditional independence test, even with sufficient sample size, will find no dependency between X and Y because TE=0 in this example. Therefore the size of the conditioning set or the sample size is not the only problem of the proposed TE-based conditional independence test, as TE will always be zero in this counter example.

   Response 1.11: Let us remind as above that this is only a problem when one wishes to interpret TE as causality. The result is completely correct for conditional dependence (statistical, as per Comment 1.1), which is what TE is; X and Y are indeed conditionally independent of eachother, given their pasts.

   This only becomes a problem when one desires the conditional dependence structure to reflect the causal structure. Considering when this can be expected to occur is the focus of this paragraph in the Discussion though, so at this point in the document the reviewer's comment is fair. As per the previous response, we thought this limitation was clear from our statement about the requirement of ideal conditions, but have modified the text to try to make this more clear again (note that faithfulness is the condition the reviewer refers to above):

   Updated text 1.2: The extent to which our method can be validated in this manner on larger more latent-confounded networks, and more importantly the extent to which the network models it infers correlate with underlying structure outside of such idealised conditions including faithfulness (see Sec. IV D), remain open questions though.

   [Reviewer in round 2] Therefore, all the following points throughout the paper should be changed accordingly including the necessary assumptions, so that the claim of conditional independence hold:

   - [Reviewer in round 2] "Our approach is shown to be capable of detecting conditional independence or otherwise even in the presence of strong pairwise time-directed correlations" in the abstract

- [Reviewer in round 2] line 164 "This adapted scheme produces surrogates which conform to the correct null hypothesis of conditional independence of the present of the target and the source history, given..."
- [Reviewer in round 2] line 302: remove "weak"
- [Reviewer in round 2] line 304 "More importantly, TE can be used to test for such conditional independence"
- [Reviewer in round 2] line 311 "In this subsection, we demonstrate that the combination of the presented estimator and surrogate generation scheme is particularly adept at identifying conditional independence in the face of strong pairwise correlations on a synthetic example." → As I said before, this is a misleading sentence. The reason why the proposed method works here as a CI test is because this is a simple synthetic example that takes into account very serious assumptions, which otherwise would be violated in real datasets.
- [Reviewer in round 2] line 424 "As argued in sections I and IV B this is equivalent to a test for conditional independence." → The sentence alone is not true. It requires the above acyclycity assumption.
- [Reviewer in round 2] line 486 "This is likely due to this scheme's poor ability to identify conditional independence in the presence of pairwise correlations, as we have already seen in Sec. II C." – Both the old and the new method suffer from the same problem and are biased into detecting more independencies if the acyclicity assumption is violated.

Response 1.12: Again as per Comment 1.1, the method is by definition a test of conditional independence; most of the changes the reviewer wishes to make above are because of misconstrued interpretations that conditional independence means a causal rather than statistical interpretation, and as such these will not be changed. Similarly, as above the acyclicity assumption is only required for the conditional independence to match causal structure – the claims referred to above are only about (statistical) conditional independence, not whether it matches causal structure.

We've removed "weak" on line 302.

Furthermore, we have added additional caveats to the 2nd last paragraph of the Discussion regarding future work investigating how the inferred conditional dependence structure may relate to underlying structure in larger, more latent-confounded networks.

[Reviewer in round 2] To conclude, I believe that the TE estimator the authors propose is a novel, important contribution that overcomes time-districtization. I am not convinced about its use as a conditional independence test, though. Therefore, I would recommend acceptance of the paper, only given that the above points became clear, the authors clearly stated the necessary assumptions and made milder the mis-formulated statements as suggested above.

Response 1.13: We thank the reviewer for the recommendation to accept. As per Comment 1.1, the points of clarity all revolve around a misconception of conditional dependence and we believe these have been clarified here.

2 Reviewer 2

We would like to thank the reviewer for supporting publication of the manuscript.

3 Reviewer 3

We would like to thank the reviewer for their constructive and useful feedback throughout the review process. This paper has become better due to their comments.

In response to their minor suggestions:

[Reviewer in round 2] Line 58: I think "as mentioned above" sounds better here

Response 3.1: We agree. This has been changed.

[Reviewer in round 2] Line 199-201: may require rewriting for better readability

Response 3.2: We agree that it did read a little awkwardly.

Updated text 3.1: This sentence has been updated to: " The emphasis of these sections is on properties of the estimators in isolation, as opposed to when combined with a statistical test. As such, we focus on the estimators' bias, variance and consistency (see Methods). "

[Reviewer in round 2] Line 499-500: I would propose to mention Fig.8b (right) for pointing out directly to the part of the figure you are commenting on.

Response 3.3: This figure reference has been updated to be more specific.

4 Reviewer 4

We would like to thank the reviewer for their comments, which have identified areas in which the clarity of our message could be improved.

Before addressing the reviewer's comments point-by-point, we would like to make a more general observation. The majority of the reviewer's comments are criticising the idea that continuous-time estimators would be expected to perform better than discrete-time estimators in many circumstances. Examples of this include their suggestion that we just need to use "minimum" bin sizes, their comment that all data is measured at some discrete precision and their assertion that undersampling is merely a "practical problem". We would encourage the reviewer to consider the related case of the estimation of information-theoretic quantities on continuous-valued data. This was historically done by discretising these continuous values and using the plugin estimator. However, since the advent of the KSG estimator [9], it has become widely accepted that estimation in *continuous space* is far preferable to discretisation (see, for example, the introduction to [18] and pages 44 to 49 of [24]). The KSG estimator is wildly popular; the original paper has received over 2500 citations and there is now a very substantial body of work extending and improving the original estimator. It is important to realise that the move from discrete space to continuous space (which the KSG estimator made possible for mutual information) is mechanistically incredible similar to the move from discrete time to continuous time. In both cases, we are moving from estimating probabilities by counting the frequencies of the occurrence of values in bins, to estimating probability densities using nearest neighbour techniques. More importantly, all the comments the reviewer makes concerning estimation in discrete time vs continuous time would, if true, apply to estimation in discrete space vs continuous space. In short, the logical extension of the reviewer's comments is that continuous-space estimators (like the KSG), cannot be expected to perform better than estimators that perform discretisation. However, this is generally accepted to not be the case.

We do, however, take the reviewer's comments seriously where some important points are raised. As such, we respond to these points individually below.

[Reviewer in round 2] Based on the previous review, the authors have made considerable new attempts to improve the quality of the paper over the previous version. For example, we appreciate the fact that they have tested their model in a number of cases. However, we still feel that there is a bias in the way they make the case for the excellence of the continuous TE method, and a balanced expression will need to be chosen. In particular, the two major remaining issues are as follows.

- [Reviewer in round 2] It is an undeniable fact that the paper didn't evaluate discrete TEs at minimum bin sizes after all, with talking about the practical problem of "the memory and computational cost of the PC they are using". Nevertheless, it is very strange that the paper says in the introduction and throughout the paper that discrete TEs produce more biased results than continuous TEs in any cases. I will discuss about this point in more detail at response 4.5.

  Response 4.1:

  The reason for not using very small bins is that this catastrophically reduces the performance of the discrete-time estimator. In the remainder of this comment we present the results of experiments demonstrating this fact.

  In order to be able to reduce the bin size while maintaining the same history length (measured by time, therefore increasing the number of bins in the history embeddings), we modified our implementation of the discrete-time estimator to more efficiently only store information on the

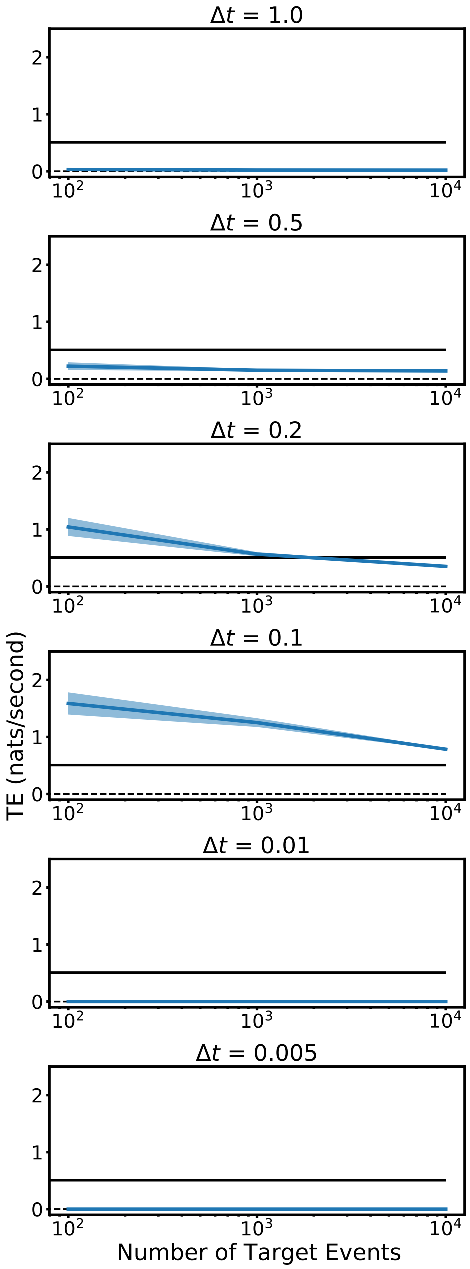

(a) TE

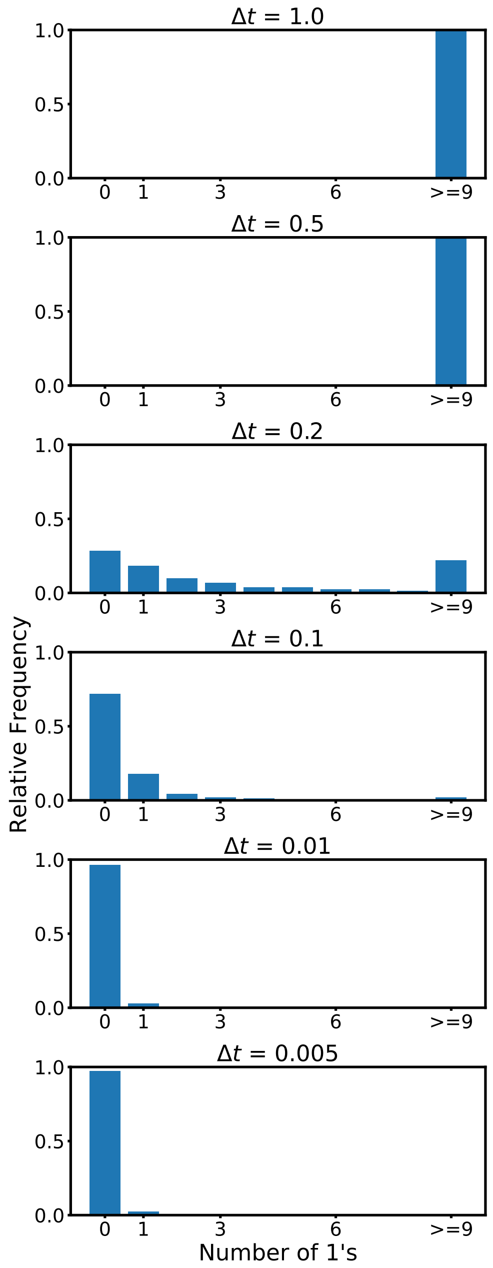

(b) Relative frequency of the number of observations of $x_t = 1$ associated with each observed history combinations $\{\mathbf{x}_{t-m:t-1}, \mathbf{y}_{t-m:t-1}\}$ for a sample size including 10^4 target spikes.

Figure 1: The discrete-time estimator run with small bins and long histories. The TE values in a for $\Delta t = [1.0, 0.5, 0.2, 0.1]$ are as per Fig 4c of the paper; $\Delta t = [0.01, 0.005]$ are added here

observed history embeddings as opposed to all possible embeddings. (This is as per the algorithmic enhancements discussed at Response 4.12). This prevented the growth in the number of possible history embeddings causing us to run out of memory.

We then reran the experiments with the discrete estimator in Results B (shown in Figure 4c of the paper). However, we added runs with very small bin sizes: 0.01 and 0.005 time units or 10x and 20x smaller than previously. We also did not truncate the history. Specifically, we used sufficient bins m in each process's history embedding $\mathbf{x}_{t-m:t-1}$ and $\mathbf{y}_{t-m:t-1}$ such that the embedding would extend over 1 time unit (the known length of the history dependence of the process). Therefore, for the smallest bin size of 0.005 time units, the history embeddings consisted of $m = 200$ bins in each process. These extremely long history embeddings reflect our statements in the Introduction and Discussion (and in the previous response to this reviewer) that small bin sizes will necessitate very long history embeddings in order to capture relevant spikes in the past, and our corresponding expectation that their large state space is likely to be dramatically undersampled in most realistic data sets.

Expectations aside, the results of these experiments are shown here in Figure 1a. We see that at the very small bin sizes (and, therefore, long history embeddings) requested by the reviewer, the estimated TE is 0, in contrast to the established ground truth of 0.5076.

It is worthwhile investigating why this occurs. Two key terms in the calculation are $p(x_t = 1 \,|\, \mathbf{x}_{t-m:t-1}, \mathbf{y}_{t-m:t-1})$ and $p(x_t = 0 \,|\, \mathbf{x}_{t-m:t-1}, \mathbf{y}_{t-m:t-1})$ (where $p(x_t = 1 \,|\, \mathbf{x}_{t-m:t-1}, \mathbf{y}_{t-m:t-1})$ is proportional to the conditional spike rate). $p(x_t = 1 \,|\, \mathbf{x}_{t-m:t-1}, \mathbf{y}_{t-m:t-1})$ is estimated by counting the number of times we observe $x_t = 1$ out of all of the times we observe the joint histories $\{\mathbf{x}_{t-m:t-1}, \mathbf{y}_{t-m:t-1}\}$. $p(x_t = 0 \,|\, \mathbf{x}_{t-m:t-1}, \mathbf{y}_{t-m:t-1})$ is simply $1 - p(x_t = 1 \,|\, \mathbf{x}_{t-m:t-1}, \mathbf{y}_{t-m:t-1})$. Now, since the spike rate in this example process never drops below 0.5 (c.f. Fig 4a), for any given joint history, the underlying true value of $p(x_t = 1 \,|\, \mathbf{x}_{t-m:t-1}, \mathbf{y}_{t-m:t-1})$ will never be 0. Similarly, the underlying true value of $p(x_t = 0 \,|\, \mathbf{x}_{t-m:t-1}, \mathbf{y}_{t-m:t-1})$ will never be 1. As such, a necessary condition for accurate estimates of $p(x_t = 1 \,|\, \mathbf{x}_{t-m:t-1}, \mathbf{y}_{t-m:t-1})$ and $p(x_t = 0 \,|\, \mathbf{x}_{t-m:t-1}, \mathbf{y}_{t-m:t-1})$ in this example is the observation of $x_t = 1$ at least one time for the combination $\{\mathbf{x}_{t-m:t-1}, \mathbf{y}_{t-m:t-1}\}$. This condition provides a very simple check for the occurence of undersampling, because in its absence we would incorrectly infer $p(x_t = 1 \,|\, \mathbf{x}_{t-m:t-1}, \mathbf{y}_{t-m:t-1}) = 0$ and $p(x_t = 0 \,|\, \mathbf{x}_{t-m:t-1}, \mathbf{y}_{t-m:t-1}) = 1$ due to not having sufficient samples of the given joint history $\{\mathbf{x}_{t-m:t-1}, \mathbf{y}_{t-m:t-1}\}$.

We explore this simple check in Figure 1b, plotting a histogram of the number of times we observe $x_t = 1$ for all of the sampled $\{\mathbf{x}_{t-m:t-1}, \mathbf{y}_{t-m:t-1}\}$ combinations. This is shown for each different bin size, when 10 000 target spikes were being used. We see that, for very small bin sizes (and therefore long histories, measured by the number of bins), almost every joint history combination has zero occurances of $x_t = 1$ associated with it. A similar process occurs for the estimation of $p(x_t = 1 \,|\, \mathbf{x}_{t-m:t-1})$ and $p(x_t = 0 \,|\, \mathbf{x}_{t-m:t-1})$. Therefore, at nearly every sample, the estimator will be (incorrectly) concluding that there is a zero probability of a spike ($x_t = 1$) when conditioning on either the target or the joint histories, due to undersampling. Whilst the reviewer's assertion that not every possible value of $\{\mathbf{x}_{t-m:t-1}, \mathbf{y}_{t-m:t-1}\}$ will be sampled (nor needs to be) is correct, this in no way obviates the massive undersampling that occurs with the discrete estimator with very small bin widths Δt. This is what leads to a TE estimate of 0.

This example highlights the pervasive and intractable nature of the undersampling problem. For sufficiently small bin sizes and long histories it is impossible to accurately estimate the conditional probability of a spike occurring. Put simply, we only have a finite number of spikes, whereas the number of possible history embeddings is growing as we decrease the bin size (while fixing the length of time the history covers). The number of history embeddings we observe out of these also generally increases (at least until we have separated each sample with $x_t = 1$ to be associated with a unique joint history $\{\mathbf{x}_{t-m:t-1}, \mathbf{y}_{t-m:t-1}\}$), so continuing to reduce the bin size serves to spread those samples with $x_t = 1$ over a larger and larger space with an inevitable

undersampling at sufficiently small bin sizes.

It is worth emphasizing here that these results show why the experiments in the paper do not use incredibly tiny bins recommended by the reviewer. We limit our bins to reasonable sizes because **this helps the performance of the discrete-time approach**. We are trying our utmost to show the discrete-time approach in as positive a light as possible. Using "minimum" bin sizes requested by the reviewer, as just demonstrated, leads to the discrete-time estimator being completely non-functional. Please see Response 4.1, as referred to again where the reviewer provides more detail on the previous response 4.5. Undersampling is already very much present at $\Delta t = 0.1$ at reasonable data-set sizes – this can be seen from the inflated TE values themselves in Figure 1a and explained by the relative frequencies in Figure 1b.[1] As such, on this example, going below $\Delta t = 0.1$ can only be expected to reduce the performance of the discrete-time estimator and so we do not believe it is productive to go below this bin size in the paper. The same logic drives our selection to avoid minimal bin sizes in the other examples.

- [Reviewer in round 2] The authors did not answer the question of what "true TE"is, which they use as the correct answer to the assessment. Please specify the formula for defining that "true TE". Without that definition, we cannot verify whether the evaluation through the mathematical model is correct or not.

Response 4.2: The definition of the continuous-time formulation of TE for spike trains is given, and discussed at length, on lines 91 to 124 (including equations (3) and (4)) of the first submission, on lines 93 to 127 (including equations (3) and (4)) in the second submission and on lines 109 to 145 (including equations (3) and (4)) of the third submission. This section of the paper has only received minor changes since the first submission.

We suspect from the new comments that the reviewer's concern appears to be that the continuous-time and discrete-time definitions are different (c.f. "If they perform estimation with a formula similar to the formula used to define it ... this is an argument that is close to tautology"). This, however, is not the case. In fact, whilst the definitions provided as above are in continuous time, what is deeply important is that (as per lines 111-114 in second submission) the original discrete time definition is equivalent as per Spinney et al. [22] – in the appropriate limit $\Delta t \to 0$, with corresponding appropriate embeddings. Without taking this limit (or bringing it down to the temporal resolution of the data, as per Response 4.10), the discrete time TE remains sensitive to the value of Δt (as originally shown by Ito et al [6]) incurring the problems discussed at those sections.

Similarly, the reviewer appears to be confusing the definition of a statistical quantity with an estimator of it. As per the above, because the two definitions (in continuous time or discrete time as $\Delta t \to 0$) are equivalent, then consistent estimators for each formalism will converge to the same value with infinite data. This assumes that $\Delta t \to 0$ is being used, which is possible with infinite data – there are major problems with such an approach though with only finite data (see Response 4.1). Indeed, because of these expected (and then verified) issues in working in the $\Delta t \to 0$ limit with the discrete-time estimator (further explored at Response 4.10 and Response 4.1), we work towards an estimator following the continuous-time formalism given the expectation that it can be more efficiently estimated (line 115-127 in second submission).

The reviewer's comments on a "true TE" concern Results part B specifically. Here, the "true value" is determined in prior work by Spinney et al. [22], from the aforementioned definition, using a numerical approach based on known conditional firing rates of the generating process, under stationary conditions. All details are available in that paper. This was referred to from line 270 in the second submission; we have added some notes to that line now. As above, this

[1] Specifically: Figure 1b shows $p(x_t = 1 \,|\, \mathbf{x}_{t-m:t-1}, \mathbf{y}_{t-m:t-1})$ is undersampled for $\Delta t = 0.1$ there, causing TE to be overestimated because $p(x_t = 1 \,|\, \mathbf{x}_{t-m:t-1})$ does not become as undersampled until smaller Δt for the same number of spikes. Moving to the smaller Δt values then also undersamples $p(x_t = 1 \,|\, \mathbf{x}_{t-m:t-1})$ causing TE to approach zero.

true value is applicable for both discrete-time and continuous-time formulations, since they are equivalent. In answer to "why don't they just use that method?" – one can only make such a direct determination of the TE from the definition when one knows the conditional firing rates; that is possible in this example where we have constructed the model / generating process ourselves. In general though, we have to estimate these from empirical data, hence the introduction of our estimator in this paper.

[Reviewer in round 1] The authors seem not to have fully considered the motivations of other researchers applying Transfer Entropy to real-world neural data, ... , what many other researchers choose to ground truth is the "true structural connectivity" and would not be a "true Transfer Entropy"...

[Response to round 1] Response 4.1: The focus of this paper is not on network inference, as per our response to all reviewers at the start of the cover letter. We agree with the reviewer that much of the community regards the inference of structural connectivity to be an important problem....

[Reviewer in round 2] The cover letter is not for the reader to read. It should be clearly stated in the introduction of the paper to the reader that network inference is not the main scope of this study.

Response 4.3: The paragraph in the Introduction discussing network inference (lines 181 to 194 of the second submission) makes it quite clear that large-scale network inference is a topic of future work and is not the focus of this paper. We have also made clear, in this paragraph and earlier in the Introduction, that what is relevant to this in our scope is validation of detection of conditional dependence where it exists (by construction) in small examples. This is an important precursor to network inference, as further explored in the Discussion.

[Reviewer in round 1] In the choice of models and parameters, there looks like to be several biases in the direction in the authors' favor. – For example, the model's parameter choices (fluctuations in firing timing, extremely strong burstiness, extremely Strong cyclicality) looks not to be natural choice.

[Response to round 1] Updated text 4.1: The new Results subsection D contains an evaluation on an example with substantially different dynamics to the examples already presented. This will be specified in more detail in response to the further points below.

[Reviewer in round 2] This point has been updated considerably. However, as indicated at the beginning of this report, no direct conclusions have been reached regarding the evaluation of the minimum bin size. Therefore, it is appropriate to use milder wording in the text.

Response 4.4: Please refer to Response 4.1 where we demonstrate the severe degradation of the performance of the discrete-time estimator with decreasing bin size. Results Section D describes how the bin size has been selected for that example to avoid the same undersampling-driven severe degredation for minimal bin size.

[Reviewer in round 1] Category 1
Some scholars, including you, have utilized Transfer Entropy's ability to quantify information flow as a property of Transfer Entropy, which is specific to information theory, but they are currently in the minority in neuroecience [22, 25, 26, 27, 32].
Category 2

The majority of researchers have utilized Transfer Entropy, as one example of many variables, to infer or compare with structural connectivity from neuronal activity [24, 28, 29, 30, 31, 31, 33, 34, 35, 36, 37].

[Response to round 1] Response 4.2: (Responding to all 3 above points) The focus of this paper is not on network inference. Please see the cover letter for more detail.

[Reviewer in round 2] Let me say it again. – If the authors state this, then the line “the focus of this paper is not network reasoning” should be written directly in the introduction, not in the cover letter.

Response 4.5: Please see response 4.3 regarding where we have stated in the Introduction as requested that dealing with network inference is left for future work.

[Response to round 1] With that said, we did indeed provide several examples (c.f. existing Result subsections C and E, and now more again with new Results subsection D) where the estimator in conjunction with the null surrogate generation method was used to test for conditional dependence/independence, in specific situations where dependence would have a one-to-one correspondence with structural connectivity. Such situations were used because they are important demonstrations of the validity of a conditional dependence/independence test, and a pre-cursor to inclusion in a more general inference algorithm. The superiority of our method over discrete time TE for inferring conditional dependence/independence (and by implication structural connectivity here) was clearly demonstrated in these examples. So, although we are not aiming at a full evaluation of incorporating our estimator into a general network inference algorithm, the head-to-head comparison with discrete-time TE for Category 2 that the reviewer wishes for is indeed present.

[Reviewer in round 2] It was unclear to me why they used different evaluation criteria for local permutation surrogate generation for continuous TE and time shifts surrogate generation for discretized TE. If the criteria were different, the accuracy would be different. I also felt that “time shifts distributed uniformly randomly between 200 and 400 time units” was unusually long. — I feel parameters and methods were not optimally selected for discrete-time TE here. — But, what does this comparison make sense?

Response 4.6: Our intention was to compare our approach (continuous-time estimation with the local permuation surrogates) against the current state-of-the art. To the very best of our knowledge, the state of the art for concluding non-zero information transfer on spike trains involves discrete-time estimation along with shifting the source in time (so as to preserve autocorrelation in the source). If a process is stationary (as assumed in TE analysis), then a longer shift will always be better for generating surrogates. Longer shifts will destroy more correlation between the source and the target, and therefore produce surrogate TE values which better represent what the TE would look like with no source-target relationship.

In any case, the reviewer is correct that we should have verified that only using permutation-based surrogates for the continuous-time estimator was not significantly affecting the results. As such, we implemented a permutation-based surrogate generation scheme for the discrete-time estimator. We reran the experiments for the discrete-time estimator using permutation-based surrogates. The results for the experiments corresponding to Figures 7b and 9d remained unchanged under this change of surrogate generation scheme. We have added short paragraphs in the text mentioning this fact. The results for the experiments corresponding to Figures 8b, S4 and S5 did change (although they remained qualitatively very similar). We have, therefore, included a new supplementary Figure S6 which displays

the results of the experiments corresponding to these figures under the change of surrogate generation scheme. We have also added a short paragraph discussing these new results in Results subsection D.

Updated text 4.1: We have added the new supplementary figure S6, showing the results of running the discrete-time estimator with a permutation-based surrogate generation scheme. We have also added short paragraphs to the text discussing which experiments had their results change under the change of surrogate generation scheme.

[Reviewer in round 2] Anyway, the authors may state in their introduction that "the focus of the paper is not on network inference", so I will not ask questions strongly here. – Tell simply where they inserted the statement in the next version.

Response 4.7: Please see response Response 4.2.

[Reviewer in round 1] An other related point is that authors will be better to state more clearly how the "true" Transfer Entropy is defined... If the method to define the so called "true" Transfer Entropy is close to that of the present authors' estimation method (whether it is a truth quantity or not), then it is not surprising that the estimated values are close each other.

[Response to round 1] Response 4.4: (Responding to all 3 above points) Transfer entropy has a true value in the same way that other statistical quantities (eg: mean or variance) have a true value, given a known generating process, stationarity, and accurate estimation of the PDFs. A principal focus in the development of any statistical estimator is that it converges to this true value.

[Reviewer in round 2] I'm very hard to understand what this statement really means. – if there are other ways of estimating True value, why don't they just use that method? The mean is the value given by the formula that defines it. If they perform estimation with a formula similar to the formula used to define it, they will get a value close to that defined value. But I am saying that this is an argument that is close to tautology.

As I asked at the beginning, first write the definition of true TE clearly. All discussion will be possible after you show it.

Response 4.8: Please see Response 4.2.

[Response to round 1] If a process naturally occurs in discrete time then one can make an argument that a discrete-time definition is natural. However, spike trains are a continuous-time phenomenon. Therefore, the discrete-time approach will only provide a natural framework for the TE rate in the limit of infinitesimal bin size. In this limit, the discrete-time approach is equivalent to the continuous-time one – i.e. the same true value exists for both estimation methods. This was demonstrated in our previous work [21].
spike trains are a continuous-time phenomenon.

[Reviewer in round 2] I feel strange about this answer. I wouldn't disagree with this claim at all if we were talking about macroscopic EEG or MEG signals. However, there is nothing disconcerting about hearing the claim that spikes are a distinctly discrete phenomenon. Neurons clearly try to hold

intervals between spikes, and discrete nature is the most prominent feature of spikes.

Response 4.9: The reviewer seems to be conflating the instantaneous (or discrete event) nature of spikes with the meaning of discrete-time processes (where time takes only discrete values), adding confusion to a simple topic. Yes, the spike events are instantaneous and have a discrete value, but although events are instantaneous (and their values are not continuous) this does not mean that they are only possible at specific values of discrete time. In contrast, spikes can in princple occur at arbitrary points in continuous time; that the spiking process is defined over a continuum of time is what makes it a continuous time process. It is well established that point processes, such as spike trains, take on discrete values in continuous time; see chapter 1 of Dayan and Abbott [4] and chapter 7 of Gerstner et. al. [5].

[Reviewer in round 2] Furthermore, it is impossible to obtain perfectly continuous data, since real-world instruments always perform discrete sampling. Thus, in describing the brain, the statement that only continuous TEs give a truth quantity is not a matter of knowing or not knowing, but of believing, at least for the moment, a matter of believing. This is also one of the reasons why the question of what is a "true TE" needs to be resolved.

Response 4.10: The reviewer is perfectly correct in stating that all real-world instruments perform discrete sampling; this is as true for measurements of time (i.e. spike times) as it is for measurements of signals in time (e.g. activity levels in fMRI/MEG/EEG).

In preparing this manuscript, we hadn't adequately considered the cases where the data is collected with poor temporal precision. In such instances, we agree that it might be feasible to run the discrete-time estimator without any further loss in precision. It is plausible that, in some cases, there may be sufficient data to accurately estimate the probability of spiking given all of the possible history permutations. In such instances, the discrete-time approach will perform as well as any TE estimation method can. We have added the following to the text in our Introduction to reflect this fact:

Updated text 4.2: Some recordings of the activities of neurons are done with low time precision. For example, recordings from calcium imaging experiments usually use a sampling rate of around 1 to 10 Hz [38]. In such cases, we could use bin sizes on the order of the experimental precision and still capture a reasonable history length with history embeddings composed of only a small number of bins. This might keep the size of the history state space small enough that we can collect an adequate number of samples for each history permutation with the available data. In such cases, we expect the discrete-time approach to perform as well as can be expected given the limitations imposed by the apparatus. On the other hand, data from microelectrode arrays can be sampled at rates over 70 kHz [39]. When using data collected with this high temporal precision, if we use bin sizes corresponding to the sampling rate, we will be forced to use incredibly short history embeddings in order to avoid the size of the history state space growing to a point where it can no longer be sampled.

We also added to the Discussion:

Updated text 4.3: Real-world data is of course sampled at some limited resolution; this means that any estimator cannot detect TE in the underlying process associated with smaller time scales than available in the data, though the consistency property of our continuous-time estimator means that it will converge to the TE value of the process at the available resolution. Of course, as per our Introduction, where temporal resolution in recordings is very poor (such as in calcium imaging experiments)

the aforementioned trade-offs for the discrete-time estimator are likely to be less problematic and the advantages of the continuous-time estimator less pronounced.

As per the 2nd new paragraph above, the fact remains that the continuous-time estimator has a consistency property which means that it will converge to the TE value of the process at the available resolution. As per the 1st new paragraph, the discrete-time estimator will only be consistent where the bin size corresponds to the sampling rate. However, spike-train data from electrode arrays is collected with very high temporal precision (as discussed in the 1st new paragraph). On this type of data, utilising the tiny bins to enable the discrete-time estimator to be consistent has a catastrophic impact on its performance, as demonstrated in Response 4.1. Therefore, practitioners are **forced** to use larger bins, rendering the estimator inconsistent.

As above, the issue of discrete sampling is an issue for any signal measurement. As such there is a vast literature on estimators of statistical quantities defined on continuous-valued variables [12], including for information-theoretic quantities, such as kernel and KSG (nearest neighbour) estimators, which are widely used e.g. for analysis of continuous-valued fMRI/EEG/MEG signals. These estimators exist and are well-recognised as producing more efficient estimates of information-theoretic quantities on continuous-valued data in comparison to discretising the continuous data, in spite of the fact that all data ultimately results from some discrete sampling. Surely the reviewer is not suggesting that all this work on continuous-space estimators for continuous-valued data is misguided for the same reason? With most finite realistic data sizes, the underlying discrete sampling is at a resolution below that at which a discretised estimator could realistically operate because when using very small partition elements with finite time series '*'these elements may contain too few data points and estimates of the probabilities are dominated by fluctuations*" (Kaiser and Schreiber [8]).

The above motivates our approach to investigating the continuous-time estimator here as a potential improvement on the discrete-time estimator, for many of the same reasons that continuous-valued estimators are more efficient than discretised-value estimators (see Introduction). That hypothesis is not simply believed, but validated through our experiments. Again, we state that the "true TE" is not only associated with the continuous-time estimator, as per Response 4.2. We also demonstrate in reponse Response 4.1 the extreme degradation of the performance of the discrete-time approach with decreasing bin size, which is pertinent to this question.

[Reviewer in round 1] When the length of the data is shorter, there is no reason why the calculation should not be possible for $\Delta t < 0.05$. Authors will be better to do effort to show results of $\Delta t = 0.01$. Therefore, final conclusions from this study will be currently proper to be expressed in these two terms.

- When people use discrete TEs, they are better to use the shorter bin size (ideally, the inverse of sampling rate).
- Continuous TE is an efficient computation with reducing necessary computational memory than discrete TE. This solution potentially solves many issues existing in this current report simultaneously.

[Response to round 1] Response 4.5: Reponding to all of the above comments so far in "Several issues of bias inherent to the evaluation method": Time discretisation is a lossy transformation (it is impossible to reconstruct the process in full detail after discretisation). This implies that it will be impossible, in all cases, to estimate the true value of the TE after such a transformation.

[Reviewer in round 2] The argument for my criticism on this topic is essentially the same as the one I have described so far. Real-world measured data is always discrete data. Even if we utilize the bin size of the measured data as it is, is the discrete TE a loss transformation? I am not saying that continuous TEs are not better than discrete TEs. However, I am uncomfortable with the fact that the authors seem to claim in the Abstract and other most notable sections that "continuous TEs always produce more accurate results than discrete TEs," even though they have not been able to evaluate discrete TEs with minimum bin sizes, etc. See the discussion below.

Response 4.11: It is correct that utilising a bin size equal to the inherent discretisation of the measured data will not be a lossy transformation. However, for data sampled with fine temporal precision as is the usual case, it is infeasible for the discrete-time estimator to function at the corresponding small bin size. Please see Response 4.1 for a demonstration of the degradation of the discrete-time estimator with decreasing bin size.

As above in Response 4.10, consideration has been added to the Discussion of cases where the inherent discretisation is very coarse and staying in discrete time is more appropriate.

[Response to round 1] There will always be a maximum total number of bins (across all processes) that can be used. This is for two reasons. Firstly, the machine on which the analysis is running will run out of memory at a certain point.

[Reviewer in round 2] The memory problem is a practical problem that can be solved by good algorithms and computers.— Even if the information of given spike timing is highly accurate, the spike data is still a pair of sparse information (index of firing neurons and their firing timings or intervals), and the number of TE values also same as the number of spikes of the target neurons. Therefore, it is even possible that the amount of required memory may not change so dramatically if the algorithm is refined well.

Response 4.12: The reviewer is correct in that there are techniques which can be used to reduce the memory usage of the discrete-time algorithm. In practice, however, such techniques are rarely used as if one is using enough time bins to make them necessary, then one is almost surely going to be faced with a severe under-sampling problem. In order to investigate this in our setting as suggested by the reviewer, we did implement a more memory efficient algorithm as per Response 4.11 and conducted the experiments presented in Response 4.1. As shown there, even where any potential memory issue is addressed the undersampling issue dominates.

[Response to round 1] Secondly, the number of possible history permutations grows exponentially in the number of bins being used.

[Reviewer in round 2] This claim is clearly wrong. — Since the spike sequence is very sparse (mostly zero), the computational cost should be far less than exponential increase if we do simple modifications, for example, looking for 1 in a large bin and dividing the bin into smaller bins only at that point, etc. I don't know if this idea is the best one, but at the end of the day, effective sampling is also a practical problem. As mentioned at the beginning of this review we should understand that the practical problems are a different level issue than the problem that a theory itself is good or bad.

Response 4.13: The claim is not wrong, the number of *possible* history permutations, 2^m for a history length m, grows exponentially in the number of bins m being used to embed that history. The number

of history embeddings which are actually observed in the data does not scale in this way, which is the point the reviewer is making in the remainder of the paragraph.

On that point then, we agree with the reviewer that this proposed approach represents a fruitful direction of research towards improving the estimation of TE on spike trains. In fact, this is *precisely* the same intuition which has motivated us in the construction of our estimator! Point processes are incredibly sparse and so it makes sense to make use of this sparseness in order to represent them more efficiently. What we have done is take this idea to its logical conclusions. The motivation for “looking for 1 in a large bin and dividing the bin into smaller bins only at that point”, is fulfilled by nearest neighbours estimators, which find an appropriate time scale for considering samples to be similar (via the k nearest neighbours distance) – for every event – given the available data.

We are very pleased that the reviewer appreciates the intuition. If the reviewer thinks that there is a better way of implementing this intuition, then we would encourage them to pursue that line of research and publish the results. We are genuinely keen to see other attempts at improving the estimation of TE on spike trains. However, in this paper we are only comparing our approach against the current state-of-the-art for discrete-time TE and are only claiming it to be superior to said state-of-the-art.

[Reviewer in round 2] This point of contention even relates to the main axis of this study. I am starting to think that using discrete and continuous TE as an opposing axis may not be a very smart way to choose the main point of view. While continuous TE seems to survey the important points accurately and efficiently, by starting from the edge of the search area, discrete TE can also find the important points with high resolution and efficiency by gradually decreasing the discretized bin size. The situation is similar to the situation in basic math analytics, where there are several ways to choose a convergent sequence of numbers to reach the limit point. So rather than saying that discrete TEs are completely bad, the statement that discrete TEs should also handle multiple scales (up to the smallest bin) seems to be more accurate expression.

Response 4.14: In some sense, the reviewer is correct on how related the two approaches are. Yet, given that a discrete-time approach forces fixed time bin width (even if one were to scan multiple widths), whilst the continuous-time approach treats time continuously and uses a different appropriate time scale for every sample (see Methods A.5 and $r_{joint,i}$ in Algorithm 1), we do feel this represents a significant distinction between the two. (This is in the same way that nearest neighbour estimators for continuous valued data such as KSG, and discrete-value estimators are related but quite distinct).

Regarding the comment on using discrete TE to examine multiple scales: Our main motiviation is in accurately estimating the true TE value and constructing a statistical test which can distinguish zero and non-zero values. We are simply searching for the methods which perform best at these tasks. Using different scales may be interesting but does not directly address these aims. We did consider adding comments on this possibility into the paper. However, given the catastrophic performance degradation at the smallest bin size requested by the reviewer in Response 4.1, we do not think it is sensible to suggest this.

[Response to round 1] Following on then, small bin widths do indeed allow one to capture more fine detail. However, they will limit the length of time over which the history can extend. In our example of the pyloric network, the simulation was conducted with a bin width of 0.01 milliseconds (the discrete-time estimator was used with a bin width of 0.05 seconds). The inter-burst intervals in this example were around 1 second. Each burst lasted around a quarter of this time. If we used a bin width of 0.01 milliseconds, this would fully capture all the time-precision of the model (this is what the reviewer seems to suggest, although they stated 0.01 sec). However, given that we have to

embed three processes, we will not be able to have these embeddings extending over longer than 0.1 milliseconds (assuming we restricted ourselves to less than 30 bins). This length of history will be woefully inadequate at capturing the dynamics of this example.

[Reviewer in round 2] The above discussion undermines all the arguments that 30 bins is the upper limit used in the premise of this subsequent verification. This point will need to be revisited.

Response 4.15: Whilst the memory is no longer the limiting factor, as per Response 4.12, the limiting factor is the extreme undersampling that occurs with long history lengths for small bin sizes. Please refer to Response 4.1 for an example demonstrating the intractability of performing estimation with minimal bin size and therefore a very large number of history bins.

[Response to round 1] Finally, to provide a concrete simulated example of this effect, we reran the experiments for Figure 4c but included bin sizes as small as 0.01. Due to our budget of 25 bins, we restricted the histories to be no longer than 12 bins. ... This is because the histories cannot cover the entire interval over which the source history is relevant. We also ran experiments without the lag described above, the results of which are worse than those shown here.

[Reviewer in round 2] From the discussion above, this logic no longer holds. If you cut the bin size in half, the number of bins would naturally have to be doubled. I'm concerned because the problem above shouldn't be too difficult when compared to the authors' skills.

Response 4.16: The reviewer's suggestion here to increase the history length with the smaller bin size would lead to history lengths of at 35 for each source and target in order to maintain the same time of history as used with our 0.05 bin width. This would result in comparably large scales of sample space (for the same number of spikes and arguably a simpler process) investigated in Response 4.1, where we show the results of running the discrete-time estimator on minimal bin sizes. We demonstrate there that this leads to a complete failure of the estimator due to extreme undersampling. As above, we designed the parameter selection here to avoid the undersampling failures, and show the discrete time estimator at its best.

[Reviewer in round 1] We are also concerned about..
P.13, L. 258
At a bin size of t = 0.2 it converges to a value less than half the true TE. Moreover, its convergence is incredibly slow. At the bin size of t = 0.1 it would appear to not have converged even after 1million target events, and indeed it is not even converging to the true value of the TE. The significance of the performance improvement by our estimator is explored further in Discussion.
The expression of not converge will be quantitatively too much strong statement (especially in the current state of limited observations). The expression of converges slowly would be more appropriate. The emphatic expression Very will be also not recommended.

[Response to round 1] Response 4.6: We disagree with the reviewer. The plots in Fig 4c clearly show that the estimate for 0.1 bin width have not converged. They are converging slowly, but in comparison to the other parameter settings and the continuous-time estimator, they have clearly not converged (dropping from 0.55 nats/s to 0.4 nats/s from NX = 105 to 106 spikes).

[Reviewer in round 2] As you can see from the previous question, unless the sampled bin size is used as it is in the discrete TE analysis, it cannot be said that we could completely compare the capabilities of continuous and discrete TEs. In Figure 4.c, the convergence destination appears to be more accurate

for the 0.1 bin width case than for the 0.2 bin width case. Furthermore, we cannot exclude the possibility that bin widths of 0.05 and 0.01[second] make the convergence more rapid. Note, however, that I am not saying that you should do the computation in short time bins. I am not suggesting that you should honestly state in the text that you have not verified the best time resolution for practical reasons.

Response 4.17: Please refer to Response 4.1 where we demonstrate the severe degradation of the performance of the discrete-time estimator with decreasing bin size. We have demonstrated there that undersampling is already occurring at 0.01 for 10^4 spikes, and becomes far worse as Δt decreases further, ruling out the possibility that smaller bin widths make the covergence more rapid.

[Reviewer in round 1] Abstract and Introduction
.. TE. Conversely, even for the discrete TE, it is possible to extract multiple time scales if we analyzed them like Wavelet analysis in comparison with Fourier transform. Let's also think the situation when keeping the bin size small and computing TEs including delays, and then people vary the degree of smoothing. Then people will be able to systematically change the focusing time scale by changing the scale and size of the time window in which you search for peaks. Solutions will exist in myriad ways.

[Response to round 1] Response 4.8:
..
The reviewer's mention of how "people will be able to systematically change the focusing time scale" highlights precisely the issue which we are trying to fix. We are proposing a method which can capture different time scales simultaneously. It produces a single estimate of the TE which captures the effects occurring on these different scales. The suggested approaches produce different estimates of the TE which capture the relationships occurring on these different time scales separately.

[Reviewer in round 2] It is very interesting that different time scales can be captured simultaneously. However, whether or not this claim makes sense depends on how "true TE" is defined.

Response 4.18: Please see response 4.2 regarding the "true TE". The point about capturing different time scales is simply that nearest neighbour estimators are able to adapt to capture both very long history dependencies in the target and more fine-grained source-target timing details – this is outlined in the Discussion paragraph "This work represents a substantial step forward ..." (and discussed further regarding nearest neighbour estimators at Response 4.13).

[Reviewer in round 1] P.5, Table4 There are several important problems with the mathematical models used in the evaluations on which all the arguments are based, and a radical solution is needed in this regard. The problems are mainly the following three points:

1. There is no explicit fluctuation in spike timing even authors gave fluctuations for intensity.
2. bursts are too strong.
3. the oscillation of membrane potential is too strong.

[Reviewer in round 2] In response to points 1-3 of the question, I thought your attempt to evaluate the model under various conditions was excellent. However, it was unclear whether the "true TE" to be

evaluated was defined in a meaningful way, and we were unable to evaluate the results of the model. As a prerequisite, I would be grateful if you could resolve this issue.

Response 4.19: Please see response 4.2.

References

[1] Nihat Ay and Daniel Polani. Information flows in causal networks. *Advances in complex systems*, 11(01):17–41, 2008.

[2] Luiz A Baccala, K Sameshima, and DY Takahashi. Generalized partial directed coherence. In *2007 15th International conference on digital signal processing*, pages 163–166. IEEE, 2007.

[3] Thomas M. Cover and Joy A. Thomas. *Elements of information theory.* John Wiley & Sons, Inc., Hoboken, NJ, USA, 2005.

[4] Peter Dayan and Laurence F Abbott. *Theoretical neuroscience: computational and mathematical modeling of neural systems.* Computational Neuroscience Series, 2001.

[5] Wulfram Gerstner, Werner M Kistler, Richard Naud, and Liam Paninski. *Neuronal dynamics: From single neurons to networks and models of cognition.* Cambridge University Press, 2014.

[6] Shinya Ito, Michael E Hansen, Randy Heiland, Andrew Lumsdaine, Alan M Litke, and John M Beggs. Extending transfer entropy improves identification of effective connectivity in a spiking cortical network model. *PloS one*, 6(11):e27431, 2011.

[7] Dominik Janzing, David Balduzzi, Moritz Grosse-Wentrup, Bernhard Schölkopf, et al. Quantifying causal influences. *The Annals of Statistics*, 41(5):2324–2358, 2013.

[8] A. Kaiser and T. Schreiber. Information transfer in continuous processes. *Physica D*, 166(1-2):43–62, 2002.

[9] Alexander Kraskov, Harald Stögbauer, and Peter Grassberger. Estimating mutual information. *Physical review E*, 69(6):066138, 2004.

[10] Solomon Kullback. *Information theory and statistics.* Courier Corporation, 1997.

[11] Purushottam W Laud and Joseph G Ibrahim. Predictive model selection. *Journal of the Royal Statistical Society: Series B (Methodological)*, 57(1):247–262, 1995.

[12] Erich L Lehmann and George Casella. *Theory of point estimation.* Springer Science & Business Media, 2006.

[13] James Massey. Causality, feedback and directed information. In *Proc. Int. Symp. Inf. Theory Applic.(ISITA-90)*, pages 303–305. Citeseer, 1990.

[14] Lukas Michelbacher, Stefan Evert, and Hinrich Schütze. Asymmetric association measures. *Proceedings of the Recent Advances in Natural Language Processing (RANLP 2007)*, 62, 2007.

[15] Judea Pearl, Madelyn Glymour, and Nicholas P Jewell. *Causal inference in statistics: A primer.* John Wiley & Sons, 2016.

[16] Jonas Peters, Dominik Janzing, and Bernhard Schölkopf. *Elements of causal inference.* The MIT Press, 2017.

[17] Jose M Quintana and Mike West. An analysis of international exchange rates using multivariate dlm's. *Journal of the Royal Statistical Society: Series D (The Statistician)*, 36(2-3):275–281, 1987.

[18] Brian C Ross. Mutual information between discrete and continuous data sets. *PloS one*, 9(2):e87357, 2014.

[19] Thomas Schreiber. Measuring information transfer. *Physical review letters*, 85(2):461, 2000.

[20] Abd-Krim Seghouane and Shun-Ichi Amari. The aic criterion and symmetrizing the kullback–leibler divergence. *IEEE Transactions on Neural Networks*, 18(1):97–106, 2007.

[21] Le Song, Mladen Kolar, and Eric Xing. Time-varying dynamic bayesian networks. *Advances in neural information processing systems*, 22:1732–1740, 2009.

[22] Richard E Spinney, Mikhail Prokopenko, and Joseph T Lizier. Transfer entropy in continuous time, with applications to jump and neural spiking processes. *Physical Review E*, 95(3):032319, 2017.

[23] Peter Spirtes, Clark N Glymour, Richard Scheines, and David Heckerman. *Causation, prediction, and search*. MIT press, 2000.

[24] Raul Vicente and Michael Wibral. Efficient estimation of information transfer. In *Directed Information Measures in Neuroscience*, pages 37–58. Springer, 2014.

[25] Daniel Yasumasa Takahashi, Luiz Antonio Baccal, and Koichi Sameshima. Connectivity inference between neural structures via partial directed coherence. *Journal of Applied Statistics*, 34(10):1259–1273, 2007.

[26] K Zografos, K Ferentinos, and T Papaioannou. Divergence statistics: sampling properties and multinomial goodness of fit and divergence tests. *Communications in Statistics-Theory and Methods*, 19(5):1785–1802, 1990.
