# Supplementary material for: Estimating Transfer Entropy in Continuous Time Between Neural Spike Trains or Other Event-Based Data
Source: PLoS Comput Biol. 2021 Apr 19;17(4):e1008054. doi: 10.1371/journal.pcbi.1008054 (PMC8084348; doi:10.1371/journal.pcbi.1008054)
Supplement: Attachment — Submitted filename: Anwer_to_Reviewer11_updated.docx [file pcbi.1008054.s011.docx]

Reviewer 4

**Based on the previous review, the authors have made considerable new attempts to improve the quality of the paper over the previous version. For example, we appreciate the fact that they have tested their model in a number of cases. However, we still feel that there is a bias in the way they make the case for the excellence of the continuous TE method, and a balanced expression will need to be chosen.　In particular, the two major remaining issues are as follows**

1. **It is an undeniable fact that the paper didn't evaluate discrete TEs at minimum bin sizes after all, with talking about the practical problem of "the memory and computational cost of the PC they are using”. Nevertheless, it is very strange that the paper says in the introduction and throughout the paper that discrete TEs produce more biased results than continuous TEs in any cases. I will discuss about this point in more detail at response 4.5.**
2. **The authors did not answer the question of what "true TE" is, which they use as the correct answer to the assessment. Please specify the formula for defining that "true TE". Without that definition, we cannot verify whether the evaluation through the mathematical model is correct or not.**

- The authors seem not to have fully considered the motivations of other researchers applying Transfer Entropy to real-world neural data, … , what many other researchers choose to ground truth is the “true structural connectivity” and would not be a “true Transfer Entropy”...

Response 4.1: The focus of this paper is not on network inference, as per our response to all reviewers at the start of the cover letter. We agree with the reviewer that much of the community regards the inference of structural connectivity to be an important problem. …

**The cover letter is not for the reader to read. It should be clearly stated in the introduction of the paper to the reader that network inference is not the main scope of this study.**

- In the choice of models and parameters, there looks like to be several biases in the direction in the authors’ favor. – For example, the model’s parameter choices (fluctuations in firing timing, extremely strong burstiness, extremely Strong cyclicality) looks not to be natural choice.

Updated text 4.1: The new Results subsection D contains an evaluation on an example with substantially different dynamics to the examples already presented. This will be specified in more detail in response to the further points below.

**This point has been updated considerably. However, as indicated at the beginning of this report, no direct conclusions have been reached regarding the evaluation of the minimum bin size. Therefore, it is appropriate to use milder wording in the text.**

Category 1

Some scholars, including you, have utilized Transfer Entropy’s ability to quantify information flow as a property of Transfer Entropy, which is specific to information theory, but they are currently in the minority in neuroecience [22, 25, 26, 27, 32].

Category 2

The majority of researchers have utilized Transfer Entropy, as one example of many variables, to infer or compare with structural connectivity from neuronal activity [24, 28, 29, 30, 31, 31, 33, 34, 35, 36, 37]. …

Response 4.2: (Responding to all 3 above points) The focus of this paper is not on network inference. Please see the cover letter for more detail.

**Let me say it again. -- If the authors state this, then the line "the focus of this paper is not network reasoning" should be written directly in the introduction, not in the cover letter.**

With that said, we did indeed provide several examples (c.f. existing Result subsections C and E, and now more again with new Results subsection D) where the estimator in conjunction with the null surrogate generation method was used to test for conditional dependence/independence, in specific situations where dependence would have a one-to-one correspondence with structural connectivity. Such situations were used because they are important demonstrations of the validity of a conditional dependence/independence test, and a pre-cursor to inclusion in a more general inference algorithm. The superiority of our method over discrete time TE for inferring conditional dependence/independence (and by implication structural connectivity here) was clearly demonstrated in these examples. So, although we are not aiming at a full evaluation of incorporating our estimator into a general network inference algorithm, the head-to-head comparison with discrete-time TE for Category 2 that the reviewer wishes for is indeed present.

**It was unclear to me why they used different evaluation criteria for local permutation surrogate generation for continuous TE and time shifts surrogate generation for discretized TE. If the criteria were different, the accuracy would be different.　I also felt that "time shifts distributed uniformly randomly between 200 and 400 time units" was unusually long. --- I feel parameters and methods were not optimally selected for discrete-time TE here. --- But, what does this comparison make sense?**

**Anyway, the authors may state in their introduction that "the focus of the paper is not on network inference", so I will not ask questions strongly here. -- Tell simply where they inserted the statement in the next version.**

- An other related point is that authors will be better to state more clearly how the “true” Transfer Entropy is defined. … If the method to define the so called “true” Transfer Entropy is close to that of the present authors’ estimation method (whether it is a truth quantity or not), then it is not surprising that the estimated values are close each other.

Response 4.4: (Responding to all 3 above points) Transfer entropy has a true value in the same way that other statistical quantities (eg: mean or variance) have a true value, given a known generating process, stationarity, and accurate estimation of the PDFs. A principal focus in the development of any statistical estimator is that it converges to this true value.

**I'm very hard to understand what this statement really means. -- if there are other ways of estimating True value, why don't they just use that method? The mean is the value given by the formula that defines it. If they perform estimation with a formula similar to the formula used to define it, they will get a value close to that defined value. But I am saying that this is an argument that is close to tautology.**

**As I asked at the beginning, first write the definition of true TE clearly. All discussion will be possible after you show it.**

If a process naturally occurs in discrete time then one can make an argument that a discrete-time definition is natural. However, spike trains are a continuous-time phenomenon. Therefore, the discrete-time approach will only provide a natural framework for the TE rate in the limit of infinitesimal bin size. In this limit, the discrete-time approach is equivalent to the continuous-time one – i.e. the same true value exists for both estimation methods. This was demonstrated in our previous work [21].

- spike trains are a continuous-time phenomenon.

**I feel strange about this answer. I wouldn't disagree with this claim at all if we were talking about macroscopic EEG or MEG signals.　 However, there is nothing disconcerting about hearing the claim that spikes are a distinctly discrete phenomenon. Neurons clearly try to hold intervals between spikes, and discrete nature is the most prominent feature of spikes.**

**Furthermore, it is impossible to obtain perfectly continuous data, since real-world instruments always perform discrete sampling.　Thus, in describing the brain, the statement that only continuous TEs give a truth quantity is not a matter of knowing or not knowing, but of believing, at least for the moment, a matter of believing.**

**This is also one of the reasons why the question of what is a "true TE" needs to be resolved.**

When the length of the data is shorter, there is no reason why the calculation should not be possible for ∆t < 0.05. Authors will be better to do effort to show results of ∆t = 0.01. Therefore, final conclusions from this study will be currently proper to be expressed in these two terms.

- When people use discrete TEs, they are better to use the shorter bin size (ideally, the inverse of sampling rate).
- Continuous TE is an efficient computation with reducing necessary computational memory than discrete TE. This solution potentially solves many issues existing in this current report simulta- neously.

Response 4.5: Reponding to all of the above comments so far in “Several issues of bias inherent to the evaluation method”:

Time discretisation is a lossy transformation (it is impossible to reconstruct the process in full detail after discretisation). This implies that it will be impossible, in all cases, to estimate the true value of the TE after such a transformation.

**The argument for my criticism on this topic is essentially the same as the one I have described so far. 　Real-world measured data is always discrete data. Even if we utilize the bin size of the measured data as it is, is the discrete TE a loss transformation?**

**I am not saying that continuous TEs are not better than discrete TEs.　However, I am uncomfortable with the fact that the authors seem to claim in the Abstract and other most notable sections that "continuous TEs always produce more accurate results than discrete TEs," even though they have not been able to evaluate discrete TEs with minimum bin sizes, etc.**

**See the discussion below.**

- There will always be a maximum total number of bins (across all processes) that can be used. This is for two reasons. Firstly, the machine on which the analysis is running will run out of memory at a certain point.

**The memory problem is a practical problem that can be solved by good algorithms and computers.--- Even if the information of given spike timing is highly accurate, the spike data is still a pair of sparse information (index of firing neurons and their firing timings or intervals), and the number of TE values also same as the number of spikes of the target neurons. Therefore, it is even possible that the amount of required memory may not change so dramatically if the algorithm is refined well.**

- Secondly, the number of possible history permutations grows exponentially in the number of bins being used.

**This claim is clearly wrong. --- Since the spike sequence is very sparse (mostly zero), the computational cost should be far less than exponential increase if we do simple modifications, for example, looking for 1 in a large bin and dividing the bin into smaller bins only at that point, etc. I don't know if this idea is the best one, but at the end of the day, effective sampling is also a practical problem.**

**As mentioned at the beginning of this review we should understand that the practical problems are a different level issue than the problem that a theory itself is good or bad.**

**This point of contention even relates to the main axis of this study.**

**I am starting to think that using discrete and continuous TE as an opposing axis may not be a very smart way to choose the main point of view.**

**While continuous TE seems to survey the important points accurately and efficiently, by starting from the edge of the search area, discrete TE can also find the important points with high resolution and efficiency by gradually decreasing the discretized bin size.**

**The situation is similar to the situation in basic math analytics, where there are several ways to choose a convergent sequence of numbers to reach the limit point.**

**So rather than saying that discrete TEs are completely bad, the statement that discrete TEs should also handle multiple scales (up to the smallest bin) seems to be more accurate expression.**

Following on then, small bin widths do indeed allow one to capture more fine detail. However, they will limit the length of time over which the history can extend. In our example of the pyloric network, the simulation was conducted with a bin width of 0.01 milliseconds (the discrete-time estimator was used with a bin width of 0.05 seconds). The inter-burst intervals in this example were around 1 second. Each burst lasted around a quarter of this time. If we used a bin width of 0.01 milliseconds, this would fully capture all the time-precision of the model (this is what the reviewer seems to suggest, although they stated 0.01 sec). However, given that we have to embed three processes, we will not be able to have these embeddings extending over longer than 0.1 milliseconds (assuming we restricted ourselves to less than 30 bins). This length of history will be woefully inadequate at capturing the dynamics of this example.

**The above discussion undermines all the arguments that 30 bins is the upper limit used in the premise of this subsequent verification.　　This point will need to be revisited.**

- Finally, to provide a concrete simulated example of this effect, we reran the experiments for figure 4c but included bin sizes as small as 0.01. Due to our budget of 25 bins, we restricted the histories to be no longer than 12 bins. … This is because the histories cannot cover the entire interval over which the source history is relevant. We also ran experiments without the lag described above, the results of which are worse than those shown here.

**From the discussion above, this logic no longer holds. If you cut the bin size in half, the number of bins would naturally have to be doubled.　I'm concerned because the problem above shouldn't be too difficult when compared to the authors' skills.**

We are also concerned about..

P.13, L. 258

At a bin size of t = 0.2 it converges to a value less than half the true TE. Moreover, its convergence is incredibly slow. At the bin size of t = 0.1 it would appear to not have converged even after 1million target events, and indeed it is not even converging to the true value of the TE. The significance of the performance improvement by our estimator is explored further in Discussion.

The expression of not converge will be quantitatively too much strong statement (especially in the current state of limited observations). The expression of converges slowly would be more appropriate. The emphatic expression Very will be also not recommended.

Response 4.6: We disagree with the reviewer. The plots in Fig 4c clearly show that the estimate for 0.1 bin width have not converged. They are converging slowly, but in comparison to the other parameter settings and the continuous-time estimator, they have clearly not converged (dropping from 0.55 nats/s to 0.4 nats/s from NX = 105 to 106 spikes).

**As you can see from the previous question, unless the sampled bin size is used as it is in the discrete TE analysis, it cannot be said that we could completely compare the capabilities of continuous and discrete TEs. 　In Figure 4.c, the convergence destination appears to be more accurate for the 0.1-bin width case than for the 0.2-bin width case.　Furthermore, we cannot exclude the possibility that bin widths of 0.05 and 0.01[second] make the convergence more rapid. Note, however, that I am not saying that you should do the computation in short time bins. I am not suggesting that you should honestly state in the text that you have not verified the best time resolution for practical reasons.**

Abstract and Introduction

… TE. Conversely, even for the discrete TE, it is possible to extract multiple time scales if we analyzed them like Wavelet analysis in comparison with Fourier transform. Let’s also think the situation when keeping the bin size small and computing TEs including delays, and then people vary the degree of smoothing. Then people will be able to systematically change the focusing time scale by changing the scale and size of the time window in which you search for peaks. Solutions will exist in myriad ways.

Response 4.8:

…

The reviewer’s mention of how “people will be able to systematically change the focusing time scale” highlights precisely the issue which we are trying to fix. We are proposing a method which can capture different time scales simultaneously. It produces a single estimate of the TE which captures the effects occurring on these different scales. The suggested approaches produce different estimates of the TE which capture the relationships occurring on these different time scales separately.

**It is very interesting that different time scales can be captured simultaneously.**

**However, whether or not this claim makes sense depends on how "true TE" is defined.**

P.52., Table4

There are several important problems with the mathematical models used in the evaluations on which all the arguments are based, and a radical solution is needed in this regard. The problems are mainly the following three points:

1. There is no explicit fluctuation in spike timing even authors gave fluctuations for intensity.

2. bursts are too strong.
3. the oscillation of membrane potential is too strong.

**In response to points 1-3 of the question, I thought your attempt to evaluate the model under various conditions was excellent.**

**However, it was unclear whether the "true TE" to be evaluated was defined in a meaningful way, and we were unable to evaluate the results of the model.　　　As a prerequisite, I would be grateful if you could resolve this issue.**
